# The case for a rational genome-based vaccine against malaria

**DOI:** 10.3389/fmicb.2014.00741

**Published:** 2015-01-22

**Authors:** Carla Proietti, Denise L. Doolan

**Affiliations:** Infectious Diseases Program, QIMR Berghofer Medical Research InstituteBrisbane, QLD, Australia

**Keywords:** malaria, vaccine, rational vaccine design, antigen discovery, genome-based, protective immunity

## Abstract

Historically, vaccines have been designed to mimic the immunity induced by natural exposure to the target pathogen, but this approach has not been effective for any parasitic pathogen of humans or complex pathogens that cause chronic disease in humans, such as *Plasmodium*. Despite intense efforts by many laboratories around the world on different aspects of *Plasmodium* spp. molecular and cell biology, epidemiology and immunology, progress towards the goal of an effective malaria vaccine has been disappointing. The premise of rational vaccine design is to induce the desired immune response against the key pathogen antigens or epitopes targeted by protective immune responses. We advocate that development of an optimally efficacious malaria vaccine will need to improve on nature, and that this can be accomplished by rational vaccine design facilitated by mining genomic, proteomic and transcriptomic datasets in the context of relevant biological function. In our opinion, modern genome-based rational vaccine design offers enormous potential above and beyond that of whole-organism vaccines approaches established over 200 years ago where immunity is likely suboptimal due to the many genetic and immunological host-parasite adaptations evolved to allow the *Plasmodium* parasite to coexist in the human host, and which are associated with logistic and regulatory hurdles for production and delivery.

## INTRODUCTION

Vaccines are the most efficient health care intervention for preventing morbidity and mortality and improving public health, except for water sanitation (World Health Organization [WHO], 2014a). The field of vaccinology originated in 1796 when Edward Jenner protected James Phipps against smallpox by inoculation with cowpox (Jenner, 1798; Baxby, 1999; Tuells, 2012). However, despite dedicated efforts to develop vaccines against a range of viral, bacterial, or parasitic diseases, approximately one third of all deaths (at least 15 million people each year) and 68% of deaths in children under 5 years of age (5 million children each year) are due to infectious diseases (World Health Organization [WHO], 2014b). Of these, three pathogens that cause chronic infections – the *Plasmodium* parasite, human immunodeficiency virus (HIV) and *Mycobacterium tuberculosis* (TB); known by public health officials as the “big three” – are the major threats responsible for 10% of all deaths globally and more than half the global burden of infectious diseases. Moreover, although there is an extensive vaccine portfolio against viral and bacterial pathogens, there are no licensed vaccines for any parasitic infection of humans or for any chronic infections by complex pathogens (World Health Organization [WHO], 2006; Moorthy and Kieny, 2010). Indeed, there is only one therapeutic vaccine approved by the US Food and Drug Administration (FDA) or European Medicines Agency (EMA), for a metastatic hormone-refractory prostate cancer (Provenge^®^, DendreonCorp, USA) but this requires preparation of a personalized vaccine for each patient and so is expensive (∼$US93,000) and has very poor uptake. New approaches for the development of vaccines against complex and chronic pathogens are urgently needed. Malaria is an excellent model for such approaches, being a complex pathogen which causes chronic infections and one of the “big three” public health targets.

The Malaria Vaccine Technology Road Map was published in 2006 as the result of a collective effort by the malaria vaccine community ^1^. A comprehensive update to this roadmap was released in 2013 with the strategic goal to, by 2030, license vaccines targeting *Plasmodium falciparum* and *P. vivax* with a protective efficacy of at least 75% against clinical malaria with a duration of protection of at least 2 years and booster doses to be required no more frequently than annually ^2^. In the intervening years, there has been a call for global malaria eradication, issued by Bill and Melinda Gates in October 2007 (Roberts and Enserink, 2007) and taken up the malaria community with a consensus community-based Malaria Eradication Agenda ^3^. Many experts consider that vaccines will play a key role in the eradication process (Hall and Fauci, 2009; Plowe et al., 2009) and vaccines will certainly be important to sustain and improve on levels of control achieved by other interventions such anti-malarial drugs, insecticide spraying and insecticide-impregnated bed nets. Additionally, the Global Vaccine Action Plan 2011–2020 compiled by stakeholders from the global health community including the World Health Organization (WHO), the United Nations Children’s Fund (UNICEF), the National Institute of Allergy and Infectious Diseases (NIAID), and the Bill & Melinda Gates Foundation (BMGF) included as a key indicator of progress towards one of its six strategic objectives, “proof of concept for a vaccine that shows greater or equal to 75% efficacy for HIV/AIDS, tuberculosis or malaria” by 2020 (World Health Organization [WHO], 2012).

However, despite intense effort for many decades by researchers throughout the world, the development of a malaria vaccine that meets the stated goals remains elusive (Sherman, 2009). Almost all efforts have focused on the pre-erythrocytic or asexual blood stages of *P. falciparum* although recent efforts have been also directed to the sexual stage to provide ‘herd immunity’ rather than confer individual protection as a consequence of the malaria eradication agenda, as well as *P. vivax* (Schwartz et al., 2012; World Health Organization [WHO], 2014b). An efficacious pre-erythrocytic vaccine is considered ideal since it would halt the development of the parasite in the liver stage and thereby prevent the blood stage of the life cycle which is the stage associated with the development of clinical symptoms, as well as the transmission of the disease which occurs in the sexual stage. The most advanced malaria vaccine candidate is RTS,S, a hybrid virus-like particle containing the C-terminus of the *P. falciparum* circumsporozoite protein (CSP) fused to hepatitis B surface antigen, expressed in *Saccharomyces cerevisiae* together with hepatitis B surface antigen (Ballou and Cahill, 2007). Recently, regulatory approval for RTS,S/ASO1 targeted at infants aged 6–14 weeks and administered through the routine Expanded Program on Immunization (EPI) has been submitted to the European Medicines Agency by GlaxoSmithKline following pivotal Phase III evaluation at 11 sites in sub-Saharan Africa; if the required regulatory approvals are obtained and the Phase III safety and efficacy data are satisfactory, the WHO has indicated that a policy recommendation for RTS,S may be granted in 2015 ^4^. Although modeling estimates predict that this vaccine should have a public health impact in terms of number of “deaths averted” (Brooks et al., 2012; Nunes et al., 2013), the Phase III efficacy of the vaccine for its intended outcome in the target age group is very low and is not sustained (Duncan and Hill, 2011; Olotu et al., 2013). Specifically, after 18 months of follow-up, efficacy against clinical malaria was only 27% among infants aged 6–12 weeks at the first vaccination; among children aged 5–17 months at first vaccination, vaccine efficacy was 46%; moreover, these results were achieved on top of existing malaria interventions including insecticide-treated bed nets used by 86 and 78% of trial participants aged 6–12 weeks or 5–17 months, respectively ^5^. Although the RTS,S data are encouraging, this milestone has taken almost 30 years of extensive preclinical and clinical development by GSK in partnership with the US Army, supported by more than US$200 million from the Bill & Melinda Gates Foundation in addition to more than $350 GSK funds to date, with an additional $260 million investment anticipated^5^ (Ballou and Cahill, 2007).

## CHALLENGES FOR MALARIA VACCINE DEVELOPMENT

The development of an effective vaccine against malaria has been hindered by the complexity of the *Plasmodium* spp. parasite as well as the host response to the parasite. Recent evidence indicates that the *Plasmodium* parasite has co-evolved with the human host for 1000s of years (Liu et al., 2010) with evolutionary co-adaptation allowing for chronic persistence of the parasite in the human host, and recurrent infections (Pierce and Miller, 2009). The *Plasmodium* life cycle involves both invertebrate (mosquito) and vertebrate (mammalian) hosts, with multiple stages within the host (sporozoite, liver, asexual blood stage, sexual) and numerous intracellular and extracellular environments in which the parasite develops (Langhorne et al., 2008). Different host responses are required to target these distinct life cycle stages – primarily antibodies against the extracellular parasite stages exposed in the peripheral circulation (sporozoites, asexual blood stage merozoites) and T cells against the intracellular stages (hepatic/liver stages) which are not accessible to circulating antibodies. In addition, the *Plasmodium* spp. parasite has a large 23 megabase genome that contains an estimated 5,300 putative proteins, many of which are expressed in different stages of the life cycle (Gardner et al., 2002). Moreover, many of these protein may exhibit allelic polymorphism (more than one allele existing for certain regions of a protein, e.g., MSP1 or AMA1), antigenic polymorphism (point mutations at the nucleotide level, e.g., CSP) often localized to B cell or T cell epitopes, or antigenic variation (multi-copy variant surfaces antigens, e.g., var genes, rifins; Good et al., 1988; Takala and Plowe, 2009; Kirkman and Deitsch, 2012; Barry and Arnott, 2014). A vaccine must be effective against all antigenic or allelic variants responses, to ensure efficacy against all variant circulating strains in the field (Moorthy and Kieny, 2010). In addition to this selection by the parasite of mutations that avoid the host protective immune response and confer susceptibility to the disease, there is selection by humans of mutations that confer resistance. Indeed, malaria is considered to have had the greatest impact of all pathogens in shaping the human genome with evidence that a number of genetic polymorphisms within the human genome, including α*-thalassemia* and hemoglobinopathies such as the sickle-cell trait, have arisen due to evolutionary pressure exerted by the *Plasmodium* parasite (Mackinnon and Marsh, 2010; Taylor et al., 2013). The high mortality rate associated with malaria would be predicted to exert a powerful selective pressure on the human genome by positively selecting any genetic mutation that confers protection against death to allow the *Plasmodium* parasite to persist in the host and be transmitted (Pierce and Miller, 2009; Mackinnon and Marsh, 2010). Other sophisticated immune evasion strategies at the host-parasite interface include the ability of the parasite to modulate the host immune response, reviewed extensively elsewhere (Langhorne et al., 2008; Casares and Richie, 2009; Pierce and Miller, 2009). These complex host–parasite interactions at the genetic and immunological levels pose significant challenges for vaccine development.

## FEASIBILITY OF VACCINATION AGAINST MALARIA

In spite of these challenges, there is growing evidence supporting the feasibility of developing an effective malaria vaccine. Field studies have demonstrated a decreasing incidence and density of infection with age and exposure to natural infection, and reduced frequency and severity of clinical illness, indicative of the acquisition of anti-disease immunity (Baird, 1998; Doolan et al., 2009). Also, passive transfer of polyclonal sera or purified immunoglobulin from individuals with lifelong exposure to *P. falciparum* resulted in a significant reduction in blood-stage parasitemia and recovery from clinical symptoms (Cohen et al., 1961; Sabchareon et al., 1991). Those studies implicate antibodies directed against blood stage antigens as the key immune effectors in naturally acquired immunity. This protection is anti-disease immunity but not anti-parasite immunity since most individuals with long-term exposure in malaria endemic areas who have developed effective clinical immunity will nonetheless continue to experience low-density, asymptomatic infections (Okell et al., 2009). Longitudinal studies in malaria-endemic populations suggest that immune responses to the pre-erythrocytic stages probably have limited involvement in this anti-disease immunity and that immunity to the pre-erythrocytic stage is not naturally acquired (Owusu-Agyei et al., 2001; Tran et al., 2013). Nonetheless, epidemiological studies suggest that exposure to low numbers of sporozoites, although not sterilizing, can reduce the parasite load in the liver and lower blood stage parasitemia, since the intensity of exposure to biting infectious mosquitoes (entomological inoculation rate) has been significantly associated with the incidence and density (but not prevalence) of *P. falciparum* parasitemia in children (Doolan et al., 2009).

More convincing evidence for the feasibility of vaccination against malaria exists from studies focused on the experimental induction of protective immunity, which have established that sterile infection-blocking protective immunity directed against the pre-erythrocytic stage can be achieved in mice and humans. Considered for many years the “gold standard” for malaria vaccine development, sterile protection can be induced in mice, non-human primates and humans by exposure to the bites of radiation-attenuated *P. yoelii, P. berghei, P. knowlesi*, or *P. falciparum* infected mosquitoes, or by intravenous immunization with isolated irradiated sporozoites, provided that the dose of radiation is sufficient to attenuate the parasite such that it can invade the hepatocyte but not develop into the blood-stage (Nussenzweig et al., 1967; Gwadz et al., 1979; Hoffman et al., 2002; Weiss and Jiang, 2012). The parasite is arrested in early liver stage development, with each invading sporozoite giving rise to only a single hepatic parasite. Murine and non-human primate studies establish that the protective immunity induced by immunization with radiation attenuated sporozoites (RAS) is directed against the liver stage parasite and mediated primarily by CD8^+^ T cells and IFN-γ (Schofield et al., 1987; Doolan and Hoffman, 2000; Tsuji, 2010; Weiss and Jiang, 2012). Recent studies have shown that protection in humans can be induced in a dose-dependent manner by intravenous but not intradermal immunization with radiation-attenuated, aseptic, purified, cryopreserved *P. falciparum* sporozoites (Epstein et al., 2011; Seder et al., 2013); five intravenous doses of 135,000 *Pf*SPZ (675,000 sporozoites in total) were required to achieve sterile immunity in 6/6 volunteers. CD8^+^ IFN-γ-producing T cells in the liver were implicated as the primary immune effector based on non-human primate studies showing that high frequencies of CD8^+^ T cells could be induced by intravenous but not intradermal routes of immunization, and mouse studies showing that these cells could protect (Epstein et al., 2011). These data are consistent with an earlier proposal (Langhorne et al., 2008) that sporozoites injected intravenously can enter the liver within seconds and be processed and presented by liver-resident antigen-presenting cells for induction of host immunity, whereas sporozoites inoculated intradermally via mosquito bite may take minutes to hours to enter the liver; or might may be taken up by a different type of antigen presenting cell such as the skin-derived CD103^+^ dendritic cells (Bedoui et al., 2009).

Sterile immunity against *Plasmodium* sporozoite challenge can be also induced in mice by homologous immunization with infectious (live) wild type sporozoites while receiving a prophylactic regimen of chloroquine (Beaudoin et al., 1977; Orjih et al., 1982; Belnoue et al., 2004) or primaquine (Putrianti et al., 2009). This immunity is directed against the liver stage and mediated by CD4^+^ and CD8^+^ T cells, but not antibodies (Belnoue et al., 2004; Roestenberg et al., 2009). More recently, this observation has been translated to humans with the demonstration that human subjects exposed three times to the bites of 10-15 *P. falciparum* infected mosquitoes under the cover of chemoprophylaxis (ChemoProphylaxis and Sporozoites, CPS-immunization; also known as infection–treatment–vaccination, ITV) were sterilely protected against subsequent challenge with *P. falciparum* sporozoites, but not *P. falciparum-*infected erythrocytes (Roestenberg et al., 2009; Bijker et al., 2013, 2014). This protection was sustained for up to 2 years (Roestenberg et al., 2011) and was dose dependent: complete protection was obtained in 4/5, 8/9, and 5/10 volunteers immunized three times with bites from 15, 10, or 5 *P. falciparum*-infected mosquitoes, respectively, and CPS immunization is thus estimated to be about 20 times more efficient than RAS immunization (Bijker et al., 2014). Since chloroquine kills asexual blood stage parasites but not sporozoites or liver stage parasites, in the CPS-model parasite infection is aborted in the early phase of blood-stage infection allowing full liver-stage development of the parasite. Consequently, CPS immunization exposes the host to parasite antigens expressed in early and late liver stages as well as early blood stages (Bijker et al., 2013). However, the protective immunity appears to be directed primarily against the liver-stage of the parasite since CPS-immunized volunteers showed no evidence of protection against blood-stage challenge *in vivo* and IgG from CPS-immunized volunteers did not inhibit asexual blood-stage growth *in vitro* (Bijker et al., 2013). Moreover, this protection appears to be mediated by T cells since protected subjects had significantly higher proportions of CD4^+^ T cells expressing the degranulation marker CD107a and CD8^+^ T cells producing granzyme B after *in vitro* restimulation with *P. falciparum*-infected red blood cells (Bijker et al., 2014), and antibodies to nine antigens representing different stages of the *P. falciparum* life cycle did not predict protection (Nahrendorf et al., 2014) even though CPS-immunization induced functional antibodies against *P. falciparum* sporozoites which could inhibit sporozoite traversal through hepatocytes and liver-stage infection (Behet et al., 2014). The antigenic targets of the CPS-induced T cell mediated are not yet known but this is under investigation.

In a variation on the CPS approach, the induction of robust protective immunity by prophylactic administration of antibiotic drugs which specifically inhibit apicoplast biogenesis during exposure to intravenously or mosquito bite transmitted sporozoites was reported in the *P. berghei* murine model (Friesen et al., 2010). This approach was conceived to overcome limitations of ITV associated with drug resistance parasite populations. The correct choice of antibiotic in this model allows for continued liver-stage maturation and exponential expansion of attenuated liver-stage merozoites from a single sporozoite and subsequent release into the host peripheral circulation of merosomes (detached vesicles containing liver stage merozoites) which are incapable of infecting red blood cells, thereby halting the parasite life cycle prior to the asexual blood stage. This immunity appears to be targeted at the liver stage and mediated primarily by CD8^+^ T cells and IFN-γ. This strategy is distinct from the RAS model where each sporozoite gives rise to only a single attenuated liver-stage parasite, and the primaquine chemoprophylaxis model where liver-stage development is aborted before the onset of nuclear divisions (Putrianti et al., 2009), and the chloroquine chemoprophylaxis model which targets the early blood-stage (Belnoue et al., 2004; Roestenberg et al., 2009).

Another area of active investigation is genetically attenuated parasites (GAP) generated via targeted disruption of genes essential for liver-stage or blood stage development (Mueller et al., 2005). These have been comprehensively reviewed elsewhere (Butler et al., 2011; Matuschewski et al., 2011; Nganou-Makamdop and Sauerwein, 2013) and include GAPs that arrest development early (Δp52/Δp36, ΔSAP1, ΔSLARP) or later (ΔUIS3/UIS4, ΔE1a, ΔE3, ΔFABI, ΔFABB/F, ΔFABZ, and ΔPKG) during liver stage development. In a first-in-human safety and immunogenicity clinical trial, 5/6 volunteers administered GAP sporozoites deleted of two *P. falciparum* pre-erythrocytic stage-expressed genes (P52 and P36) via mosquito bite did not develop blood stage parasitemia (Spring et al., 2013). However, the development of peripheral parasitemia in one volunteer showed that this double knockout GAP was incompletely attenuated. Although no breakthrough blood infections were observed in a study evaluating the *P. yoelii* Δp52/Δp36 GAP (Labaied et al., 2007), others observed developing *P. berghei* or *P. falciparum* liver stages *in vitro* culture with the respective Δp52/Δp36 GAPs and breakthrough blood infections in *P. berghei* Δp52/Δp36 GAP immunized mice, showing that the Δp52/Δp36 GAP was not adequately attenuated (Annoura et al., 2012). A minimal set of screening criteria has been proposed to assess the adequacy of genetically attenuation before advancing candidate GAPs into further clinical development (Annoura et al., 2012). Most recently, a triple gene deleted GAP (*Pf* Δp52/Δp36/Δsap1) had been shown to be completely attenuated in a humanized mouse model (Mikolajczak et al., 2014).

Vaccination with chemically attenuated parasites is also being pursued. In the original studies in mice, chemical attenuation of *P. yoelii* or *P. berghei* sporozoites with the DNA sequence-specific alkylating agent centanamycin conferred sterile immunity *in vivo* following one to three intravenous doses (50/20/20K) of centanamycin-treated *P. yoelii* or *P. berghei* sporozoites (Purcell et al., 2008a,b). The level of protection, parasite-specific antibodies, and IFN-γ-producing CD8^+^ T cell responses induced by chemically attenuated sporozoites (CAS) were similar to those induced by RAS. In the blood stage, Good et al., (2013) have recently reported that a single immunizing dose of 10^6^ *P. chabaudi* parasitized red blood cells chemically attenuated with centanamycin could protect against challenge with 10^5^ homologous or heterologous (*P. vinckei* and *P. yoelii*) parasites in a CD4^+^ T cell dependent manner (Good et al., 2013). Chemically attenuated *P. falciparum* parasitized red blood cells are currently being evaluated in the clinic (Good, personal communication).

Another whole organism based strategy directed at the blood stage of the parasite life cycle was designed to induce T helper 1 (Th1) cell mediated immunity in the absence of antibodies by immunizing with subpatent ultra-low dose parasitized erythrocytes followed by drug treatment (Pombo et al., 2002). This built on observations that parasites in high density could cause apoptosis of parasite-specific T cells (Hirunpetcharat and Good, 1998; Xu et al., 2002). Good et al. (2013) showed that malaria-naive humans deliberately infected four times with approximately 30 viable parasitized red blood cells followed by drug treatment developed robust T cell responses in the absence of antibody which prevented parasite growth in three of four individuals and delayed the onset of parasite growth which remained subpatent in the fourth individual (Pombo et al., 2002). Efficacy against a higher dose challenge post-immunization was not assessed, and the contribution of residual drug to this protection could not be excluded. This ultra-low dose immunization approach has not yet been repeated in humans or mice. However a subsequent study in the *P. chabaudi* model demonstrated that three intravenous infections with a relatively high dose of 100,000 *P. chabaudi* infected erythrocytes followed by drug cure after 48 h and before microscopic patency could protect mice against a 10-fold higher (10^6^) parasite challenge; mice had robust cell-mediated immune responses and antibodies to merozoite antigens but variant-specific antibodies were not detectable (Elliott et al., 2005).

These proof-of-concept studies with whole organism based vaccines show that experimental induction of sustained protective immunity to *Plasmodium* spp. parasites is possible.

## MALARIA VACCINE STRATEGIES

Evidence that immunity can be induced experimentally or acquired naturally with age and/or exposure suggests two fundamental approaches to vaccine development (Good and Doolan, 2010):

(1) Induce robust immune responses against a selected panel of antigens recognized as immunodominant in the context of natural infection.
(2) Induce a broad immune response against a large number of parasite antigens not necessarily recognized as immunodominant in the context of natural infection in order to mimic the immunity induced by the whole parasite.

Until recently, almost all malaria vaccine efforts have been directed at the former approach. Most of these subunit efforts have targeted only a very small number of target antigens, focusing almost exclusively on CSP for the pre-erythrocytic stage and MSP1 and AMA1 for the blood stage (Schwartz et al., 2012; World Health Organization [WHO], 2014b) and investigating a variety of vaccine delivery systems. A major emphasis has been on purified recombinant proteins formulated with adjuvant, but viral vectored approaches have become of increasing interest, particularly for the pre-erythrocytic stage where induction of parasite-specific T cell responses is desirable. These have been reviewed extensively elsewhere (Bruder et al., 2010; Crompton et al., 2010b; Anders, 2011; Schwartz et al., 2012; Birkett et al., 2013). However, despite extensive efforts throughout the world spanning many decades and in contrast to the immunity induced by experimental immunization with variations of whole organism based vaccines, candidate subunit vaccines against malaria have been poorly efficacious (Schwartz et al., 2012; World Health Organization [WHO], 2014b). Indeed, it is not surprising that a vaccine based on a single antigen is unlikely to confer solid protection against a complex multi-lifecycle stage parasite expressing thousands of proteins that has co-evolved with the human host for millennia.

This marked lack of success in single-antigen subunit based vaccines, combined with the recognition that an effective malaria vaccine will likely need to be a multi-stage multi-immune response vaccine (Doolan and Hoffman, 1997) given the challenges described above, has caused a resurgence of interest in whole organism vaccine approaches, intended to reproduce the protective immunity induced by exposure to the parasite in experimental challenge models or naturally in the field. However, a number of challenges are associated with whole organism based vaccine strategies (Menard, 2005; Ballou and Cahill, 2007; Anders, 2011). Specific concerns with GAPs include potentially inadequate attenuation, as already demonstrated with the *Pf* Δ52/Δp36 GAP in the only human trial to date (Spring et al., 2013), and reversion to virulence of a parasite that has co-evolved with the human host for millennia if it is genetically modified in only one or a few regions of its genome. Other concerns include logistical challenges associated with manual dissection of sporozoites, route of administration, loss of viability upon cryopreservation, and cold-chain requirements. Additionally, antigenic variability of the parasite means that robust cross-protection from a single strain product is essential. Thus, although promising results have been obtained in preclinical models, it remains to be seen whether the many technical, logistical, and regulatory hurdles associated with large-scale production and field deployment of live-attenuated parasites can be overcome.

Even if these technical, logistical, and regulatory challenges can be overcome, a key question is whether whole parasite approaches will induce optimal immunity. Those approaches are essentially similar to the classical “identify, isolate, and inject” approach pioneered in the late 17th century by Edward Jenner, which has proved successful with a wide range of bacterial and viral pathogens, but not yet any parasitic pathogens or any chronic diseases (reviewed in Doolan et al., 2014). This could be attributed to the complexity of parasites as compared to viruses and bacteria, with larger genomes and multiple intracellular and extracellular life cycle stages. Additionally, it is now well established that microbial pathogens have evolved complex and efficient ways of counteracting and evading innate and adaptive immune mechanisms of the host (Zepp, 2010). Thus, logically, robust immunity against such pathogens would not be induced by strategies using the whole pathogen intended to mimic experimentally that immunity induced by natural exposure. Rather, effective vaccination would require that we do better than nature, by inducing responses that are quantitatively and/or qualitatively different immune response to that induced by natural infection.

Inherent in this approach is the cumulative effect of multiple potentially low level immune responses directed against a number of antigens which may or may not be dominant parasite antigens, which together exceed a response threshold sufficient to protect. We proposed this “threshold of immune response concept” over 15 years ago (Doolan and Hoffman, 1997). Specifically, we proposed that the intensity of an immune response will be determined by the sum of a number of signals received by a T cell (or B cell) with the appropriate receptor, and that although a single antigen (with one or more target epitopes) could be sufficient to generate a protective immune response if it is appropriately presented to the immune system, a wide repertoire of specificities at the epitope level (more antigens) should increase the probability of collectively inducing a protective host immune response. Experimental evidence that responses to a given antigen following protective immunization in mice with RAS are not as high as antigen-specific responses induced by vaccination with antigen-specific peptides, recombinant protein or live vectors which nonetheless fail to protect validates this concept. In an elegant series of studies in the *P. berghei* model, Harty and colleagues were able to define a threshold frequency of CD8^+^ T cells that predicted long-term sterile immunity against sporozoite challenge, and showed that an extremely high frequency of CD8^+^ T cells (exceeding 8% of all circulating CD8^+^ T cells in BALB/c mice and 19% in outbred mice) was required for both a single-antigen subunit vaccination (Schmidt et al., 2008) and RAS immunization (Schmidt et al., 2010); this level greatly exceeding the number of memory CD8^+^ T cells required for resistance to other pathogens.

A similar requirement for a protective threshold of antibody production for parasite clearance following lethal challenge has been demonstrated in a *P. chabaudi* model using MSP1-specific transgenic CD4^+^ T cells in immunodeficient mice, where levels of MSP1-specific antibody and the speed of their production correlated with the time of resolution of infection (Stephens et al., 2005). In humans, in the field, a broad repertoire of antibody responses to multiple antigens has been associated with protection from clinical malaria (Osier et al., 2008; Crompton et al., 2010a).

These data suggest that to induce optimal protection against malaria, vaccination with the whole parasite is not required, and would likely be suboptimal. Rather, vaccination with only key components of the parasite that have been rationally selected in the context of relevant biological function would be preferable.

## NOT ALL ANTIGENS ARE EQUAL

It is now generally recognized that not all antigens, or epitopes within a given antigen, are equal in the context of host immunity. The phenomenon whereby immune responses are mounted against only one or a few of the entire repertoire of peptide epitopes expressed on a given antigen, or antigens expressed by a given pathogen, is termed immunodominance (Sercarz et al., 1993; Akram and Inman, 2012). In theory, any of the proteins expressed by the parasite genome may be a target of protective immune responses. However, many proteins expressed by the parasite genome do not elicit immune responses, and for many if not most of the subset which do elicit immune responses, the response is not protective. Also in theory, a robust and effective immune response directed against an accessible dominant target would be highly successful in eliminating the pathogen from the host. However, as noted above, in many cases immune evasion strategies have evolved to allow the pathogen to escape the protective host response. Factors that could influence immunodominance, and their importance in protection, have not been investigated in the context of a complex pathogen, although some hypotheses exist (Doolan et al., 2014).

Although immunization with whole organisms preferentially induces responses against immunodominant epitopes, responses to subdominant T cell epitopes can contribute to controlling infection (Friedrich et al., 2007; Kloverpris et al., 2009; Ruckwardt et al., 2010). Also, the ability to focus the immune response away from dominant antigens or epitopes and towards subdominant antigens or epitopes could be of value in chronic diseases where T cells directed against the immunodominant antigens or epitopes might be anergic but T cells specific for non-dominant epitopes might be reactive. Translating this to infectious diseases where the development of effective vaccines based on immunodominant antigens has thus far not been successful (Good and Doolan, 2010), one could speculate that the critical targets of protective immunity may be those that are not dominant in the context of the whole organism.

In support of this, although the CSP is the dominant sporozoite surface protein and represents a target of immune responses induced by immunization with radiation attenuated *Plasmodium* sporozoites, those responses are much weaker than responses induced by CSP-based subunit vaccines, and responses are also directed against other non-CSP antigens (Doolan et al., 1997, 2000, 2003; Kumar et al., 2006; Gruner et al., 2007; Trieu et al., 2011). Notably, sterile CD8^+^ T-cell mediated immunity to sporozoite challenge could be induced by immunization with RAS in JHT transgenic mice that were tolerant to CSP, so this protection was directed against non-CSP antigens (Kumar et al., 2006). Sterile protection could be also induced by RAS or CPS immunization with transgenic *P. berghei* parasites in which the endogenous CSP was replaced by that of *P. falciparum* or *P. yoelii,* respectively, despite the absence of immune responses specific to the CSP expressed by the parasite used for challenge (Gruner et al., 2007; Mauduit et al., 2010). Also, human volunteers protected by immunization with RAS did mount CD8^+^ and CD4^+^ T cell and antibody responses to CSP but those responses were similar to, or lower than, those in immunized volunteers who were not protected against sporozoite challenge indicating that the RAS-induced protective immune responses are directed predominantly against non-CSP antigens (Doolan et al., 1997, 2000, 2003; Trieu et al., 2011). Recently, using protein microarrays, we have identified a signature of 19 mostly uncharacterized antigens which is strongly associated with RAS-induced protective immunity; reactivity to any individual antigen did not correlate with protection (Trieu et al., 2011).

Accumulating experimental data in preclinical and clinical studies of malaria thus indicate that in fact not all antigens are equal, that antigen selection is important, and that it is the cumulative response to a number of key antigens that is important, rather than a dominant response to a single antigen.

## GENOME-BASED VACCINE DESIGN

The identification within the hierarchy of antigens (or epitopes) expressed by the pathogen that are targets of protective immune responses and that will stimulate effective immunity against that pathogen is a key component of rational vaccine design (Rueckert and Guzman, 2012). Cutting-edge technologies and screening strategies to mine genomic sequence information for state-of-the-art rational vaccine design, as well as genome-based rational vaccine design strategies, and recently reviewed elsewhere (Doolan et al., 2014; Schussek et al., 2014). The challenge, then, is how to select the key targets since there is no algorithm that can be applied to identify the important antigens and epitopes. Advances in the genomic era offer great potential, particularly when the genome of the target pathogen is large, and large-scale genomic, proteomic and transcriptomic datasets provide valuable resources to mine for antigen discovery.

In the case of malaria, the recent availability of large-scale genomic (**Table 1**), proteomic (**Table 2**), transcriptomic (**Table 3**) and comparative data from *P. falciparum* and other *Plasmodium* species provides an unprecedented opportunity to identify key targets antigens of protective immunity amongst the large repertoire of antigens expressed by the whole parasite. Since 2002, the genomes of seven *Plasmodium* parasites have been published, including that for the two major human parasites (*P. falciparum, P. vivax*) (Gardner et al., 2002; Carlton et al., 2008; Pain et al., 2008); two non-human primate parasites (*P. knowlesi*, *P. cynomolgi*; Pain et al., 2008; Tachibana et al., 2012), and three murine parasites (*P. yoelii* 17XNL, *P. berghei, P. chabaudi*; Carlton et al., 2002; Hall et al., 2005). Draft complete genomes are also available for the avian malaria parasite *P. gallinaceum*, non-human primate parasite *P. reichenowi* Denni, the lethal murine parasite *P. yoelii YM,* and three different strains of *P. falciparum* (HB3,Dd2 and IT; **Table 1**). Partial genome sequence is also accessible for 21 isolates of *P. falciparum* and four isolates of *P. vivax* from geographically distinct areas of the world, as well as low coverage draft genomes for the other two parasites infecting humans, *P. malaria* and *P. ovale*. With the advent of next-generation sequencing technology, the sequencing of genomes for an additional 105 *Plasmodium* species/strains/isolates have been proposed by the malaria community ^6^. These parasites include 50 *P. falciparum* field isolates collected from patients in East Africa, America, and Asia; 24 *P. falciparum* parasites representing both contemporary or historical parasite strains, including strains used in drug and vaccine trials; 16 *P. vivax* isolates from Africa, America, and Asia; four non-human primate parasites (*P. reichenowi, P. cynomolgi*, *P. inui*, *P. coatneyi*, *P. fragile*); and complete sequence and closure of three murine parasites (*P. chabaudi*, *P. yoelii*, and *P. berghei* and two avian and reptile parasites (*P. relictum* and *P. mexicanum*). For some of these parasites, a first partial assembly is already available (**Table 1**).

**Table 1:** *Plasmodium* genomic datasets.

Genome	Strain	Host	Submitter	Status	Reference
*P. falciparum*	*3D7*	Human	Genome Sequencing Consortium	High quality genome produced in 2002. Updated and reassembled version available on GeneDB (September 2011)	Gardner et al. (2002); GeneDB; PlasmoDB; Parasite Genomics Group (WTSI)
*P. falciparum*	*IT*	Human	WTSI*	Draft genome produced using Sanger sequencing and Illumina sequence-by-synthesis. Annotation version available on GeneDB (March 2013)	GeneDB; PlasmoDB, Parasite Genomics Group (WTSI)
*P. falciparum*	*HB3, Dd2*	Human	Broad Institute	Draft genomes available on NCBI^$^ (September 2009)	NCBI
*P. falciparum*	*20 strains*	Human	Broad Institute	First-pass partial assemblies available on NCBI for *7G8, CAMP/Malaysia, Vietnam Oak-Knoll (FVO) D10, D6, FCH/4, IGH-CR14, K1, MaliPS096_E11, NF135/5.C10, NF54, Palo Alto/Uganda, RAJ116, RO-33, Santa Lucia, Senegal_V34.04,Tanzania, UGT5.1, VS/1, Brasil I.*	NCBI
*P. vivax*	*Sal-1*	Human	TIGR	Published in 2008. Updated and reassembled version (10x coverage) available on GeneDB (May 2013)	Carlton et al. (2008); GeneDB; PlasmoDB; Parasite Genomics Group (WTSI)
*P. vivax*	*India VII, Mauritiana, North Korean*	Human	Broad Institute	First-pass partial assembly available on NCBI (July 2012)	NCBI
*P. malariae*		Human	WTSI	Partial draft genome	Parasite Genomics Group (WTSI)
*P. ovale*		Human	WTSI	Low-coverage draft produced using Sanger sequencing, from multiple sources of *P. ovale* DNA (Nigeria I/CDC strain and LSHTM)	Parasite Genomics Group (WTSI)
*P. knowlesi*	*H*	Primate and Human	WTSI	Published in 2008. Updated and reassembled version (8× coverage) available on GeneDB (March 2014)	Pain et al. (2008); GeneDB; PlasmoDB; Parasite Genomics Group (WTSI)
*P. cynomolgi*	*B*	Primate	Osaka University	Draft genome obtained using Illumina Sequence-by-synthesis technology. Updated contig sequence and annotation available on PlasmoDB (September 2013)	Tachibana et al. (2012); PlasmoDB, Parasite Genomics Group (WTSI)
*P. reichenowi*	*CDC Dennis*	Primate	WTSI	First-pass partial assembly produced using Sanger sequencing available on NCBI (May 2014)	Parasite Genomics Group (WTSI).
*P. inui*	*San-Antonio1*	Primate	Broad Institute	First-pass partial assembly available on NCBI (January 2014)	NCBI
*P. coatneyi*	*Hackeri*	Primate	NHGRI	First-pass partial assembly available on NCBI (July 2014).	NCBI
*P. gaboni*	*Pgk*	Chimpanzee	TIGR**	First-pass partial assembly available on NCBI (February 2014)	NCBI
*P. gallinaceum*	*8A*	Avian	WTSI	Low-coverage draft genome produced by Sanger sequencing. Updated high quality draft genome is being produced using Illumina Sequence-by-synthesis.	Parasite Genomics Group (WTSI)
*P. yoelii yoelii*	*17X and 17XNL*	Murine	WTSI	Low-coverage draft genome of 17XNL published in 2002. Updated and reassembled version of 17X available on GeneDB (May 2013)	Carlton et al. (2002); Hall et al. (2005); GeneDB; PlasmoDB; Parasite Genomics Group (WTSI)
*P. yoelii yoelii*	*YM*	Murine	WTSI	First-pass partial assembly available on GeneDB (January 2012)	GeneDB; Parasite Genomics Group (WTSI)
*P. chabaudi*	*chabaudi*	Murine	WTSI	Low-coverage draft genome published in 2005. Additional sequencing completed using Illumina Sequence-by-synthesis technology and available on GeneDB (March 2013)	Hall et al. (2005); GeneDB; PlasmoDB. Parasite Genomics Group (WTSI)
*P. berghei*	*Anka*	Murine	WTSI	Low-coverage draft genome published in 2005. Additional sequencing completed using Illumina Sequence-by-synthesis technology and available on GeneDB (March 2013)	Hall et al. (2005); GeneDB; PlasmoDB; Parasite Genomics Group (WTSI)
*P. vinckei*	*Petteri, Vinkei*	Murine	Broad Institute	First-pass partial assembly available on NCBI (2014)	NCBI

*WTSI, Wellcome Trust Sanger Institute; **TIGR, The Institute for Genomic Research; ^$^NCBI, National Center for Biotechnology Information

**Table 2:** *Plasmodium* proteomic datasets.

Species/Strain	Parasite material	Method	Description	Reference
*P. falciparum 3D7*	Infected erythrocytes and gametocytes	MudPIT*	*P. falciparum* asexual blood stage and sexual stage proteomes. 2,415 parasite proteins identified.	Florens et al. (2002)
*P. falciparum* NF54	Trophozoites, schizonts, gametocytes, and gamete	LC-MS/MS**	*P. falciparum* asexual blood stage and sexual stage proteomes. 1,289 proteins detected: 714 in asexual blood stages, 931 in gametocytes and 645 in gametes.	Lasonder et al. (2002)
*P. falciparum* 3D7	Infected erythrocytes	MudPIT	*P. falciparum* parasite infected erythrocyte surface proteome. 423 proteins identified.	Florens et al. (2004)
*P. falciparum 3D7*	Infected erythrocytes	MudPIT	*P. falciparum* infected erythrocyte proteome. 802 proteins identified in the nuclear proteome.	Oehring et al. (2012)
*P. falciparum 3D7 and P. vivax Sal-1*	Infected erythrocytes	LC-MS/MS	Proteomic analyses of clinical isolates of early stages of *P. falciparum* and *P. vivax*. 100 proteins identified.	Acharya et al. (2009)
*P. falciparum 3D7, F12*	Trophozoites, gametocytes	LC-MS/MS	Quantitative comparative proteomics analysis of trophozoites and early gametocyte stages of *P. falciparum*. 1090 new proteins identified.	Silvestrini et al. (2010)
*P. vivax Sal-1*	Infected erythrocytes	MS/MS^†^	Elucidation of the *P. vivax s*chizont proteome from clinical sample. 316 proteins identified.	Roobsoong et al. (2011)
*P. falciparum 3D7 and P. berghei ANKA*	Midgut and salivary glands Sporozoites	nLC-MS/MS	Proteomic comparison of sporozoites from oocysts and salivary glands. 127 proteins identified in oocysts, 450 in oocyst-derived sporozoites, and 477 in salivary gland sporozoites.	Lasonder et al. (2008)
*P. falciparum 3D7 and P. yoelii 17XNL*	Salivary gland sporozoites	LTQ Orbitrap Velos§, nLC-MS/MSi	Putative surface proteomes of *P. falciparum* and *P. yoelii* salivary gland sporozoites. 1991 *P. falciparum* sporozoite proteins and 1876 *P. yoelii* sporozoite proteins identified.	Lindner et al. (2013)
*P. berghei ANKA*	Mixed asexual blood stages, gametocytes, ookinetes, oocysts and salivary gland sporozoites	MudPIT	*P. berghei* oocysts and sporozoite proteomes. 1836 proteins identified: 1139 in blood stage, 1091 in ookinetes, 733 in gametocytes, 277 in oocysts and 134 in salivary gland sporozoites.	Hall et al. (2005)
*P. berghei ANKA*	Gametocytes	LC-MS/MS	Comparative proteomic analysis of male vs female gametocytes. 353 proteins in mixed-gametocyte proteome identified, 305 proteins in male gametocytes, and 170 proteins in female gametocytes.	Khan et al. (2005)
*P. yoelii 17X*	Infected hepatocytes	LC-MS/MS	*P. yoelii* liver stage proteome. 712 proteins identified.	Tarun et al. (2008)

*MudPIT, Multidimensional protein identification technology; ^∗∗^LC-MS/MS, liquid chromatography tandem mass spectrometry; nLC-MS/M S, nano flow liquid chromatography mass spectrometry; ^†^MS/MS, tandem mass spectrometry; §LTQ Orbitrap Velos, TQ Orbitrap Velos mass analyzer coupled to nano-liquid chromatography.

**Table 3:** *Plasmodium* transcriptomics datasets.

Species/Strain	Parasite material	Method	Description	Reference
*P. falciparum 3D7*	Nine different life cycle stages: mosquito salivary gland sporozoites, seven asexual erythrocytic stage time ponts, and sexual stage gametocytes	Microarray	Gene expression profiles of human and mosquito stages of *P. falciparum* life cycle. 43% of expressed genes were cell-cycle regulated; 1489 genes regulated in erythrocytic stages, and 746 genes differentially regulated in sporozoites and gametocytes.	Le Roch et al. (2003)
*P. falciparum 3D7*	Trophozoites and schizonts	Microarray	Gene-expression profile of the intraerythrocytic trophozoite and schizont stages. Revealed extensive transcriptional regulation of genes specialized for processes specific to trophozoites or schizonts.	Bozdech et al. (2003)
*P. falciparum 3D7*	Gametocytes stages I–V	Microarray	Transcriptomic analysis of high-purity stage I-V *P. falciparum* gametocytes. Identified a sexual development cluster of 246 genes exhibiting highly correlated, gametocyte-specific expression patterns.	Young et al. (2005)
*P. falciparum 3D7, Dd2, HB3*	Asexual erythrocytic stages	Microarray	Transcriptome of asexual intraerythrocytic developmental cycle (3D7, 6287; Dd2, 5294; HB3, 6415 genes). 60% of genome identified as transcriptionally active during erythrocytic stage. Transcripts profiles were well conserved amongst strains, except for surface antigens.	Bozdech et al. (2003) Llinas et al. (2006)
*P. falciparum:* 21 lines, from four subclonal groups (3D7A/3D7B, 7G8, D10, HB3A/HB3B)	Asexual erythrocytic stage at seven time points post-infection (10, 20, 30, 34, 37, 40, or 43 h)	Microarray	Asexual blood-stage transcriptome of 21 *P. falciparum* lines, from four subclonal groups. Transcriptional heterogeneity among essentially isogenic parasites was observed.	Rovira-Graells et al. (2012)
*P. falciparum 3D7*	Peripheral blood samples from infected patients	Microarray	*In vivo* gene expression profiles of parasites isolated from clinical samples. Expression profiles clustered into three distinct groups corresponding to distinct physiological states: glycolytic growth, starvation response, or general (non-nutritional) stress response.	Daily et al. (2007)
*P falciparum 3D7 and NF54*	Sporozoites and gametocytes(3D7 stages II, III, and V; NF54, stage V)	Quantitative PCR	Transcript profiles of *rif* and *var* genes. A single rif gene, PF13_0006, showed high transcript abundance in mature gametocyte stage V and in sporozoites.	Wang et al. (2010)
*P. falciparum* FcB1	Late schizont/merozoite stages and rings/trophozoites/early schizonts stages	ESTs	EST library of highly synchronized *P. falciparum* parasites to isolate genes selectively expressed during merozoite morphogenesis, using SSH*. Identified genes selectively expressed during the last hours of the erythrocytic cycle. Subsequently identified 243 protein coding genes, including 121 hypotheticals, by sequencing 22,125 clones from the SSH library.	Florent et al. (2004, 2009)
*P. falciparum 3D7*	Asexual erythrocytic stage at 8 time points post-infection (5, 10, 15, 20, 25, 30, 35, and 40 h)	RNA-seq	RNA-seq analysis of the transcriptome throughout intraerythrocytic development. Variation in overall transcriptional activity with stage-specific regulation (low at early stages, peaking at trophozoite stage).	Bartfai et al. (2010)
*P. falciparum 3D7*	Asexual erythrocytic stage at seven time points post-infection	RNA-seq	Illumina-based RNA seq throughout intraerythrocytic development. Identified 107 novel transcripts and 38 pseudogenes, with many demonstrating differential expression over time.	Otto et al. (2010)
*P. falciparum 3D7*	Seven life cycle stages: two gametocyte stages (II and V), ookinete, and four asexual erythrocytic stages (ring, early trophozoite, late trophozoite, and schizont	RNA-seq	Transcriptomic analysis of asexual and sexual stages. Identified many unknown splicing junctions and stage specific gene expression including occyst-specific genes.	Lopez-Barragan et al. (2011)
*P. falciparum 3D7*	Parasites cultured *in vitro*, as well as two pools of field isolates from *Plasmodium*-infected pregnant women and children	NSR-seq	Transcriptome of *in vitro* and *in vivo* blood stages. Identified a subset of genes upregulated in parasite-infected pregnant women; and a subset of genes that differentiated parasites infecting children from parasites infecting pregnant women.	Vignali et al. (2011)
*P. falciparum 3D7*	Mixed asexual erythrocytic stages	ESTs	7,683 *P. falciparum* 3D7 ESTs were generated from mixed asexual stages.	Zhang et al. (2011)
*P. falciparum 3D7*	Asexual erythrocytic stages	SAGE	Transcriptional profile of erythrocytic stages in different studies.	Patankar et al. (2001), Gunasekera et al. (2003, 2004)
*P. vivax clinical isolates*	Peripheral blood samples from infected patients	Microarray	Complete transcriptional profile throughout the intraerythrocytic cycle of three clinical isolates from acute *P. vivax* patients. Identified distinct expression patterns genes predicted to encode proteins associated with virulence and host pathogen interactions.	Bozdech et al. (2008)
*P. vivax Sal-1*	Human and mosquito stages, including sporozoites, gametes, zygotes and ookinetes, and *in vivo* asexual blood stages	Microarray	Characterization of the *P. vivax* transcriptome. Distinct stage-specific expression profiles. Identified DNA sequence motifs upstream of co-expressed genes that are conserved across different species.	Westenberger et al. (2010)
*P. yoelii yoelii 17X*	Infected hepatocytes at 3 timepoints post-infection (24, 40 and 50 hr); midgut-oocyst sporozoites and salivary gland sporozoites; and mixed blood stages and blood-stage schizonts	Microarray	Profile of genome-wide liver stage gene expression was compared with other life cycle stages. Identified 1985 genes active during liver stage development including 1000 upregulated genes and 174 genes that were more abundant or unique in the liver stage.	Tarun et al. (2008)
*P. falciparum*	cDNA from *P. falciparum* salivary gland sporozoites vs. sporozoites co-cultured with human primary hepatocytes for 1 h	Microarray	Transcriptome of salivary gland sporozoites was compared with that of sporozoites co-cultured with hepatocytes. 532 genes were up-regulated and 79 genes downregulated following co-culture, in comparison to non-exposed salivary gland sporozoites. Two proteins with temporal upregulation (PFD0425 [SIAP1] and Pf08_0005 [SIAP2]) implicated in both traversal and hepatocyte invasion.	Siau et al. (2008)
*P. yoelii yoelii 17XNL*	Midgut sporozoites and salivary gland sporozoites	Microarray	Comparative transcriptome study of *P. yoelii* oocyst sporozoites and salivary gland sporozoites. 124 genes were upregulated and 47 downregulated in salivary gland sporozoites. Similar transcription profiles of 11 *P.falciparum* orthologs confirmed by qPCR.	Mikolajczak et al. (2008)
*P. yoelii yoelii 17X*	cDNA library from *P. yoelii* liver stages laser-capture microdissected at 40 h post infection	EST	623 non-redundant genes were identified, of which 25% were unique to the liver stage.	Sacci et al. (2005)
*P. berghei HPE and HP*	Non-gametocyte producing clone (HPE) and gametocyte producing clone (HP); rings, young trophozoite, young schizont, young gametocyte, mature trophozoite, mature and mature gametocytes	Microarray	Comparative transcriptomes of non-gametocyte and p gametocyte producing clones of *P. berghei*. 215 and 355 genes were upregulated in the G1 and the S/M phases, respectively. 58% of the G1 proteins (125 genes) and 59.4% of the S/M proteins (199 genes) were also up-regulated in gametocytes.	Hall et al. (2005)
*P. falciparum 3D7, P. vivax Sal-1, P. yoelii, P. berghei ANKA*	Full-length cDNA libraries (all stages)	ESTs	Comparative transcriptomes of full-length cDNA sequences of *P. falciparum* (12,484 cDNAs), *P. vivax* (9,633 cDNAs), *P. yoelii* (11262), and *P. berghei* (1518 cDNAs).	Watanabe et al. (2007)

*SSH, suppression subtractive hybridization.

In addition to this genomic data, the rapid development of high throughput technologies for profiling the transcriptome, proteome, metabolome, and interactome, including capillary liquid chromatography, tandem mass spectrometry (LC-MS/MS), Multidimensional Protein Identification Technology (MudPIT), microarray DNA chip, yeast two-hybrid (Y2H) screening and most recently RNA seq and NSR-seq (Winzeler, 2006) can be applied for the rational identification of potential vaccine candidate antigens.

In early studies, proteomes of four stages of the *P. falciparum* parasite life cycle (sporozoites, merozoites, trophozoites, and gametocytes) were revealed by MudPIT (Florens et al., 2002) as well as the proteome of the asexual blood stages (trophozoites and schizonts) and sexual stages (gametocytes and gamete) by LC-MS/MS analysis (Lasonder et al., 2002). This *P. falciparum* sporozoite proteome included a total of 1048 proteins of which almost half (49%) were unique to this stage. The proteomes of *P. berghei* oocysts and sporozoite were subsequently defined by MudPIT in 2005, resulting in the identification of 1836 proteins (Hall et al., 2005). Recently, nano-liquid chromatography (nanoLC) coupled high-resolution MS was applied to profile the proteome of highly purified salivary gland sporozoites from *P. falciparum* and *P. yoelii*, identifying a total of 1991 *P. falciparum* sporozoite proteins and 1876 *P. yoelii* sporozoite proteins (Lindner et al., 2013).The liver stage proteome was defined in the rodent host *P. yoelii* by LC-MS/MS resulting in the detection of 712 proteins in the liver stage schizont proteome, with 174 of them more abundant and/or detected only in the liver stage (Tarun et al., 2008).

A study of *P. falciparum* infected erythrocytes, fractionated through biotin-streptavidin interaction and analyzed by MudPIT, identified 164 proteins of the 423 proteins that were enriched the in biotin-labeled fractions and thus considered surface proteins. Among these were known secreted proteins, such as Exp-1 and Exp-2, and rhoptry proteins (RAP-1, -2, and -3, RhopH-2 and -3; Florens et al., 2004). Another LC-MS/MS study of early stages from *P. falciparum* clinical isolates detected 88 *P. falciparum* proteins in the peripheral circulation (Acharya et al., 2009). More recent analyses on the proteome of asexual trophozoites, early gametocytes, and mature gametocytes from *in vitro* culture by high accuracy (LC MS/MS) have identified over new 1000 parasite proteins, not previously identified (Silvestrini et al., 2010). Most recently, proteomic analysis by MudPIT of *P. falciparum* nuclei of ring, trophozoite and schizont stages has elucidated the nuclear proteome of *P. falciparum* during intra-erythrocytic development, consisting of over 800 proteins (Oehring et al., 2012).

Elucidation of the gametocyte sex-specific proteomes of *P. falciparum* by LC-MS/MS resulted in the identification of 305 unique proteins in the male gametocyte proteome and 170 unique proteins in the female gametocyte proteome (Khan et al., 2005). The identification of sex-specific proteins has brought new insight in understanding the role of these proteins during the sexual differentiation and thus proving the basis for identifying targets for the interruption of transmission, either by drugs or vaccines.

Other comparative LC MS/MS proteomic studies of *P. falciparum* and *P. berghei* have identified novel proteins in the pre-erythrocytic stages of the *Plasmodium* life cycle: 127 proteins in the oocyst proteome, 450 proteins in oocyst-derived sporozoites and 477 proteins in salivary gland sporozoites, for a total of 728 *Plasmodium* proteins, of which 250 were exclusively detected in the oocyst/sporozoite stages when compared to the *P. falciparum* blood stage proteome (Lasonder et al., 2008).

More recently, the proteome of *P. vivax* asexual schizonts has been defined by analyzing fresh parasite isolates from patients exposed to *P. vivax* by tandem MS/MS (Roobsoong et al., 2011).

Complementing the proteomic analyses, a number of transcriptomics studies have been undertaken, ranging from analysis of gene transcription using random clones selected from genomic DNA libraries to more recent global expression transcription profile using oligonucleotide microarray, RNA-seq or NSR-seq. Transcriptomic data are now available from multiple life-cycle stages or gene knock-out mutants of *P. falciparum* and *P. berghei* as well as multiple stages of *P. yoelii* (mosquito, erythrocytic and liver stages; **Table 3**). Specifically, transcriptomic data available for *P. falciparum* include genome-scale transcriptomic analyses of nine different life cycle stages (3D7 strain) including salivary gland sporozoite, early and late ring stage, early and late trophozoite, early and late schizont, merozoite, and gametocyte stages (Le Roch et al., 2003); the intraerythrocytic trophozoite and schizont stages (Bozdech et al., 2003); the intraerythrocytic developmental cycle of *P. falciparum* HB2, Dd2, and 3D7 strains (Llinas et al., 2006); as well as 21 other *P. falciparum* lines from four subclonal groups (3D7A/3D7B, 7G8, D10, HB3A/HB3B) during the asexual intraerythrocytic developmental cycle at seven time points post-infection (10, 20, 30, 34, 37, 40, or 43 h; Rovira-Graells et al., 2012) plus analysis of parasites derived directly from blood samples from 43 infected patients (Daily et al., 2007). The transcriptome of high-purity stage I-V *P. falciparum* gametocytes is also available (Young et al., 2005), as well as the transcript profiles of rif and var genes at different stage of gametocytogenesis (Wang et al., 2010).

In addition to genome-wide gene expression studies, RNA-seq analysis of the *P. falciparum* transcriptome is available for multiple time points during the intraerythrocytic developmental cycle (Bartfai et al., 2010; Otto et al., 2010; Lopez-Barragan et al., 2011); and two gametocyte stages (Lopez-Barragan et al., 2011). The transcriptional profile of two pools of field isolates from malaria-infected pregnant women and children has been also determined by NSR-seq (Vignali et al., 2011).

For *P. vivax*, transcriptomic data include genome-scale transcriptomic analyses throughout the intraerythrocytic cycle of three distinct *P. vivax* isolates (Bozdech et al., 2008) as well as sporozoites co-cultured with hepatocytes (Siau et al., 2008); and sporozoites, gametes, zygotes, and ookinetes, and asexual blood stages obtained from infected patients (Westenberger et al., 2010).

*Plasmodium yoelii* gene expression data are available for oocyst sporozoites and salivary gland sporozoites (Mikolajczak et al., 2008); three time points during the liver stage (24, 40, and 50 h post-infection), two time points during the mosquito stage (midgut-oocyst sporozoites and salivary gland sporozoites; Tarun et al., 2008), and two intraerythrocytic stages (Tarun et al., 2008). *P. berghei* expression data are available for rings, young trophozoites, young schizonts, and mature schizonts (Hall et al., 2005).

Other transcriptomic datasets include EST data from cDNA libraries of *P. falciparum* (12,484 cDNA sequences), *P. vivax* (9,633 cDNAs), *P. yoelii* (11,262 cDNAs), and *P. berghei* (1,518 cDNAs) (Watanabe et al., 2007; Tarun et al., 2008), as well as 7,683 *P. falciparum* 3D7 ESTs generated from mixed asexual stages and SAGE data (Patankar et al., 2001; Gunasekera et al., 2003, 2004).

These genome-wide genomic, proteomic, and transcriptomics analyses (**Tables 1**–**3**) have revealed potential antigens expressed in the sporozoite and intrahepatic stages and novel proteins on the surface of malaria-infected erythrocytes that may play a role in pathogenesis and immunity, and that may represent potential new vaccine candidates.

Several novel parasite surface antigens have been discovered (Florens et al., 2002, 2004; Lasonder et al., 2002; Le Roch et al., 2004; Sam-Yellowe et al., 2004) but, so far, this wealth of data has yielded few new vaccine targets (**Table 4**; Duffy et al., 2012). In our opinion, translation of this wealth of information from the large-scale genomic, proteomic, and transcriptomic datasets into practical application, such as the identification of promising new target antigens for vaccine development, requires integrating this knowledge with functional outputs such as biologically relevant immune responses. Thus, in our laboratory, we are pursuing immunomics-based approaches which integrate the disciplines of genomics and immunology using biological samples from humans or animals with immunity to the disease of interest to identify the subset of pathogen-derived proteins or their epitopes that are recognized by the host immune system (Klysik, 2001; Doolan, 2011). No vaccines derived from immunomics have yet reached the stage of clinical testing but a number of promising candidate antigens have been identified by us in the malaria model using antibody based (Doolan et al., 2008; Crompton et al., 2010a; Trieu et al., 2011) or T-cell based (Doolan et al., 2003; Doolan, 2011). We are using peripheral blood mononuclear cells (T cells) and plasma/sera (antibodies) from individuals experimentally immunized with RAS or CPS or naturally exposed to malaria for proteome-wide immune screening assays using clinically relevant selection criteria to identify the key antigens and their epitopes recognized by recall *Plasmodium*-specific immune responses in protective human models. Data generated to date establish proof-of-concept for both T cell and antibody based approaches to identify from genomic datasets the subset of antigens and epitopes which represent promising candidates for next generation malaria vaccine development (Doolan, 2011; Matuschewski et al., 2011; Doolan et al., 2014; **Table 4**).

**Table 4:** *Plasmodium* antigens identified from genome-based datasets.

Antigen	Model	Main finding	Reference
Ag2/CelTOS	*P. falciparum* RAS T cell screening;*P. yoelii* and *P. berghei* murine immunization/challenge	One of four highly reactive *P. falciparum* proteins identified by T cell based screening of 27 putative proteins with RAS immunized volunteers; conferred cross-species protection against *P. yoelii* and *P. berghei* sporozoite challenge.	Doolan (in preparation), Doolan et al. (2003); Bergmann-Leitner et al. (2010)
Thirty-four pre-erythrocytic antigens	*P. yoelii* murine immunization/challenge	Only three antigens (P33p[*Py*52] [PY01340], Ag2 [*Py*CelTOS], and Ag5[PY00419] elicited CD8^+^ T cell responses but none conferred protection.	Mishra et al. (2011)
PY03011, PY03424, and PY03661: pre-erythrocytic antigens	*P. yoelii* murine immunization/challenge	The combination of the three antigens (but not individual antigens) conferred sterile protection against *P. yoelii* sporozoite challenge in a high proportion of mice.	Limbach et al. (2011)
PyTmp21(PY06414): pre-erythrocytic antigen	*P. yoelii* murine immunization/challenge	*Py*Tmp21 elicited functional immunity that significantly reduced liver stage parasite burden following *P. yoelii* sporozoite challenge.	Chen et al. (2014)
PbS20 and PbTRAP pre-erythrocytic antigens	*P. berghei* murine immunization/challenge	Systematically evaluated H(2b)-restricted peptides predicted from genome-wide analysis, and identified two epitopes as targets of CD8^+^ T cells induced by whole parasite vaccines; CD8^+^ T cells specific for the *Pb*TRAP epitope but not the PbS20 epitope inhibited liver stage parasite development *in vivo*	Hafalla et al. (2013)
PyTAM: blood-stage antigen	*P. yoelii* murine immunization/challenge	*Py*TAM elicited functional immunity that conferred significant protection against mortality from lethal *P. yoelii* challenge infection.	Cherif et al. (2014)
Py01157: sexual and sexual stage antigen	*P. yoelii* murine immunization/challenge	*Py*01157: conferred partial protection against challenge with non-lethal *P. yoelii* 17XNL, but not against challenge with lethal *P. yoelii* 17XL.	Zhang et al. (2012)

In the initial proof-of-concept study (Doolan et al., 2008) for the application of protein microarray technology for antigen discovery, a protein microarray with 250 pre-erythrocytic *P. falciparum* proteins were probed with sera from individuals with sterile immunity or no immunity against experimental challenge following vaccination with radiation-attenuated *P. falciparum* sporozoites, partial immunity acquired by natural exposure, or no previous exposure to *P. falciparum*, to identify 72 highly reactive P. falciparum antigens. Subsequently, a larger protein microarray containing 2,320 *P. falciparum* proteins fragments (corresponding to 1200 *P. falciparum* proteins, ∼25% of the proteome) was screened with plasma from clinically divergent groups of individuals immunized with *P. falciparum* irradiated sporozoites and identified a signature of 16 antigens strongly associated with RAS-induced sterile protective immunity; responses to any individual antigen were not associated with protection (Trieu et al., 2011). Using this same protein microarray, plasma from children and young adults naturally exposed to malaria in an endemic area of Mali were screened and 46 novel proteins significantly associated with a reduction of re-infection in the subsequent malaria season were identified (Crompton et al., 2010a). One *P. falciparum* antigen identified by T cell based screening of RAS-immunized volunteers (Doolan et al., 2003), known as Ag2 or CelTOS (Kariu et al., 2006), has been shown to be a target of cross-species protection in the murine model (Bergmann-Leitner et al., 2010). Another *P. yoelii* antigen, PyTmp1, could induce immune responses that reduced liver stage parasite burden *in vivo* (Chen et al., 2014), and three other *P. yoelii* antigens in combination (but not individually) could induce sterile immunity in a high proportion of immunized mice (Limbach et al., 2011). Two *P. yoelii* blood stage antigens, PyTAM and Py01157, have been also associated with partial protection in the murine model (Zhang et al., 2012; Cherif et al., 2014; **Table 4**). These data support the potential of genome-based antigen discovery. Assessment of the protective capacity of promising new antigens discovered by genome-based screening is currently a bottleneck in the preclinical development pipeline. Development and refinement of a high throughput screening system would overcome this obstacle and allow the development of a rationally designed genome-based vaccine against malaria.

## CONCLUSION

We advocate a modern genome-based approach to rational vaccine design which takes advantage of the wealth of genomic, proteomic, and transcriptomic datasets, using biological samples from humans or animals with immunity to the disease of interest and functionally relevant screening criteria to identify the key antigens and their epitopes targeted by protective immune responses. With this focus, we can improve on nature. In the case of malaria, validated human challenge models provide a valuable resource for genome-based antigen discovery. Moreover, this information can be integrated with other omic-based and cutting-edge immunological based approaches. For example, systems immunology could be applied to identify immune signatures that distinguish a protective immune response from a non-protective one. The many technological and intellectual advances in the omics era offer great potential for the development of rationally designed vaccines against malaria as well as other pathogens that have thus far proved elusive.

## Conflict of Interest Statement

The authors declare that the research was conducted in the absence of any commercial or financial relationships that could be construed as a potential conflict of interest.

## Footnotes

1 http://www.malariavaccine.org/files/Malaria_Vaccine_TRM_Final_000.pdf

2 http://www.who.int/immunization/topics/malaria/vaccine_roadmap/en/

3 http://www.who.int/malaria/elimination/maleraupdate.pdf

4 http://www.malariavaccine.org/files/MVI-GSK-RTSSfactsheetFINAL-web.pdf

5 http://www.malariavaccine.org/files/MVI-GSK-FAQ-FINAL-web.pdf

6 http://www.genome.gov/pages/research/der/pathogensandvectors/plasmodiumwhitepaperv8.pdf

## References

[B1] Acharya P. Pallavi R. Chandran S. Chakravarti H. Middha S. Acharya J. (2009). A glimpse into the clinical proteome of human malaria parasites *Plasmodium falciparum* and *Plasmodium vivax*. *Proteom. Clin. Appl.* 3 1314–1325 10.1002/prca.200900090 21136953

[B2] Akram A. Inman R. D. (2012). Immunodominance: a pivotal principle in host response to viral infections. *Clin. Immunol.* 143 99–115 10.1016/j.clim.2012.01.015 22391152

[B3] Anders R. F. (2011). The case for a subunit vaccine against malaria. *Trends Parasitol.* 27 330–334 10.1016/j.pt.2011.04.003 21592861

[B4] Annoura T. Ploemen I. H. van Schaijk B. C. Sajid M. Vos M. W. van Gemert G. J. (2012). Assessing the adequacy of attenuation of genetically modified malaria parasite vaccine candidates. *Vaccine* 30 2662–2670 10.1016/j.vaccine.2012.02.010 22342550

[B5] Baird J. K. (1998). Age-dependent characteristics of protection versus susceptibility to *Plasmodium falciparum.* *Ann. Trop. Med. Parasitol.* 92 367–390 10.1080/00034989859366 9683890

[B6] Ballou W. R. Cahill C. P. (2007). Two decades of commitment to malaria vaccine development: GlaxoSmithKline biologicals. *Am. J. Trop. Med. Hyg.* 77 289–295. 18165505

[B7] Barry A. E. Arnott A. (2014). Strategies for designing and monitoring malaria vaccines targeting diverse antigens. *Front. Immunol.* 5:359 10.3389/fimmu.2014.00359 PMC4112938 25120545

[B8] Bartfai R. Hoeijmakers W. A. Salcedo-Amaya A. M. Smits A. H. Janssen-Megens E. Kaan A. (2010). H2A.Z demarcates intergenic regions of the *Plasmodium falciparum* epigenome that are dynamically marked by H3K9ac and H3K4me3. *PLoS Pathog.* 6:e1001223 10.1371/journal.ppat.1001223 PMC3002978 21187892

[B9] Baxby D. (1999). Edward Jenner’s Inquiry; a bicentenary analysis. *Vaccine* 17 301–307 10.1016/S0264-410X(98)00207-2 9987167

[B10] Beaudoin R. L. Strome C. P. A. Mitchell F. Tubergen T. A. (1977). *Plasmodium berghei*: immunization of mice against the ANKA strain using the unaltered sporozoite as an antigen. *Exp. Parasitol.* 42 1–5 10.1016/0014-4894(77)90054-6 324783

[B11] Bedoui S. Whitney P. G. Waithman J. Eidsmo L. Wakim L. Caminschi I. (2009). Cross-presentation of viral and self antigens by skin-derived CD103+ dendritic cells. *Nat. Immunol.* 10 488–495 10.1038/ni.1724 19349986

[B12] Behet M. C. Foquet L. van Gemert G. J. Bijker E. M. Meuleman P. Leroux-Roels G. (2014). Sporozoite immunization of human volunteers under chemoprophylaxis induces functional antibodies against pre-erythrocytic stages of *Plasmodium falciparum*. *Malar. J.* 13 136 10.1186/1475-2875-13-136 PMC4113136 24708526

[B13] Belnoue E. Costa F. T. Frankenberg T. Vigario A. M. Voza T. Leroy N. (2004). Protective T cell immunity against malaria liver stage after vaccination with live sporozoites under chloroquine treatment. *J. Immunol.* 172 2487–2495 10.4049/jimmunol.172.4.2487 14764721

[B14] Bergmann-Leitner E. S. Mease R. M. De La Vega P. Savranskaya T. Polhemus M. Ockenhouse C. (2010). Immunization with pre-erythrocytic antigen CelTOS from *Plasmodium falciparum* elicits cross-species protection against heterologous challenge with *Plasmodium berghei*. *PLoS ONE* 5:e12294 10.1371/journal.pone.0012294 PMC2924390 20808868

[B15] Bijker E. M. Bastiaens G. J. Teirlinck A. C. van Gemert G. J. Graumans W. van de Vegte-Bolmer M. (2013). Protection against malaria after immunization by chloroquine prophylaxis and sporozoites is mediated by preerythrocytic immunity. *Proc. Natl. Acad. Sci. U.S.A.* 110 7862–7867 10.1073/pnas.1220360110 23599283 PMC3651438

[B16] Bijker E. M. Teirlinck A. C. Schats R. van Gemert G. J. van de Vegte-Bolmer M. van Lieshout L. (2014). Cytotoxic markers associate with protection against malaria in human volunteers immunized with *Plasmodium falciparum* sporozoites. *J. Infect. Dis.* 10.1093/infdis/jiu293 [Epub ahead of print]. PMC4208622 24872326

[B17] Birkett A. J. Moorthy V. S. Loucq C. Chitnis C. E. Kaslow D. C. (2013). Malaria vaccine R&D in the decade of vaccines: breakthroughs, challenges and opportunities. *Vaccine* 31(Suppl. 2), B233–B243 10.1016/j.vaccine.2013.02.040 23598488

[B18] Bozdech Z. Llinas M. Pulliam B. L. Wong E. D. Zhu J. DeRisi J. L. (2003). The transcriptome of the intraerythrocytic developmental cycle of *Plasmodium falciparum*. *PLoS Biol.* 1:E5 10.1371/journal.pbio.0000005 PMC176545 12929205

[B19] Bozdech Z. Mok S. Hu G. Imwong M. Jaidee A. Russell B. (2008). The transcriptome of *Plasmodium vivax* reveals divergence and diversity of transcriptional regulation in malaria parasites. *Proc. Natl. Acad. Sci. U.S.A.* 105 16290–16295 10.1073/pnas.0807404105 18852452 PMC2571024

[B20] Brooks A. Briet O. J. Hardy D. Steketee R. Smith T. A. (2012). Simulated impact of RTS,S/AS01 vaccination programs in the context of changing malaria transmission. *PLoS ONE* 7:e32587 10.1371/journal.pone.0032587 PMC3295753 22412892

[B21] Bruder J. T. Angov E. Limbach K. J. Richie T. L. (2010). Molecular vaccines for malaria. *Hum. Vaccin.* 6 54–77 10.4161/hv.6.1.10463 20061792

[B22] Butler N. S. Schmidt N. W. Vaughan A. M. Aly A. S. Kappe S. H. Harty J. T. (2011). Superior antimalarial immunity after vaccination with late liver stage-arresting genetically attenuated parasites. *Cell Host Microbe* 9 451–462 10.1016/j.chom.2011.05.008 21669394 PMC3117254

[B23] Carlton J. M. Adams J. H. Silva J. C. Bidwell S. L. Lorenzi H. Caler E. (2008). Comparative genomics of the neglected human malaria parasite *Plasmodium vivax*. *Nature* 455 757–763 10.1038/nature07327 18843361 PMC2651158

[B24] Carlton J. M. Angiuoli S. V. Suh B. B. Kooij T. W. Pertea M. Silva J. C. (2002). Genome sequence and comparative analysis of the model rodent malaria parasite *Plasmodium yoelii yoelii*. *Nature* 419 512–519 10.1038/nature01099 12368865

[B25] Casares S. Richie T. L. (2009). Immune evasion by malaria parasites: a challenge for vaccine development. *Curr. Opin. Immunol.* 21 321–330 10.1016/j.coi.2009.05.015 19493666

[B26] Chen L. Keitany G. J. Peng X. Gibson C. Mohar I. Vignali M. (2014). Identification of pre-erythrocytic malaria antigens that target hepatocytes for killing in vivo and contribute to protection elicited by whole-parasite vaccination. *PLoS ONE* 9:e102225 10.1371/journal.pone.0102225 PMC4099202 25025375

[B27] Cherif M. S. Shuaibu M. N. Kodama Y. Kurosaki T. Helegbe G. K. Kikuchi M. (2014). Nanoparticle formulation enhanced protective immunity provoked by PYGPI8p-transamidase related protein (PyTAM) DNA vaccine in *Plasmodium yoelii* malaria model. *Vaccine* 32 1998–2006 10.1016/j.vaccine.2014.01.005 24440206

[B28] Cohen S. McGregor I. Carrington S. (1961). Gamma-globulin and acquired immunity to human malaria. *Nature* 192 733–737 10.1038/192733a0 13880318

[B29] Crompton P. D. Kayala M. A. Traore B. Kayentao K. Ongoiba A. Weiss G. E. (2010a). A prospective analysis of the Ab response to *Plasmodium falciparum* before and after a malaria season by protein microarray. *Proc. Natl. Acad. Sci. U.S.A.* 107 6958–6963 10.1073/pnas.1001323107 20351286 PMC2872454

[B30] Crompton P. D. Pierce S. K. Miller L. H. (2010b). Advances and challenges in malaria vaccine development. *J. Clin. Invest.* 120 4168–4178 10.1172/JCI44423 21123952 PMC2994342

[B31] Daily J. P. Scanfeld D. Pochet N. Le Roch K. Plouffe D. Kamal M. (2007). Distinct physiological states of *Plasmodium falciparum* in malaria-infected patients. *Nature* 450 1091–1095 10.1038/nature06311 18046333

[B32] Doolan D. L. (2011). *Plasmodium* immunomics. *Int. J. Parasitol.* 41 3–20 10.1016/j.ijpara.2010.08.002 20816843 PMC3005034

[B33] Doolan D. L. Apte S. H. Proietti C. (2014). Genome-based vaccine design: the promise for malaria and other infectious diseases. *Int. J. Parasitol.* 44 901–913 10.1016/j.ijpara.2014.07.010 25196370

[B34] Doolan D. L. Dobano C. Baird J. K. (2009). Acquired immunity to malaria. *Clin. Microbiol. Rev.* 22 13–36 10.1128/CMR.00025-08 19136431 PMC2620631

[B35] Doolan D. L. Hoffman S. L. (1997). Multi-gene vaccination against malaria: a multistage, multi-immune response approach. *Parasitol. Today* 13 171–178 10.1016/S0169-4758(97)01040-5 15275087

[B36] Doolan D. L. Hoffman S. L. (2000). The complexity of protective immunity against liver-stage malaria. *J. Immunol.* 165 1453–1462 10.4049/jimmunol.165.3.1453 10903750

[B37] Doolan D. L. Hoffman S. L. Southwood S. Wentworth P. A. Sidney J. Chesnut R. W. (1997). Degenerate cytotoxic T cell epitopes from P. *falciparum* restricted by multiple *HLA-A* and *HLA-B* supertype alleles. *Immunity* 7 97–112 10.1016/S1074-7613(00)80513-0 9252123

[B38] Doolan D. L. Mu Y. Unal B. Sundaresh S. Hirst S. Valdez C. (2008). Profiling humoral immune responses to *P. falciparum* infection with protein microarrays. *Proteomics* 8 4680–4694 10.1002/pmic.200800194 18937256 PMC3021802

[B39] Doolan D. L. Southwood S. Chesnut R. Appella E. Gomez E. Richards A. (2000). HLA-DR-promiscuous T cell epitopes from *Plasmodium falciparum* pre- erythrocytic-stage antigens restricted by multiple HLA class II alleles. *J. Immunol.* 165 1123–1137 10.4049/jimmunol.165.2.1123 10878392

[B40] Doolan D. L. Southwood S. Freilich D. A. Sidney J. Graber N. L. Shatney L. (2003). Identification of *Plasmodium falciparum* antigens by antigenic analysis of genomic and proteomic data. *Proc. Natl. Acad. Sci. U.S.A.* 100 9952–9957 10.1073/pnas.1633254100 12886016 PMC187898

[B41] Duffy P. E. Sahu T. Akue A. Milman N. Anderson C. (2012). Pre-erythrocytic malaria vaccines: identifying the targets. *Expert Rev. Vaccines* 11 1261–1280 10.1586/erv.12.92 23176657 PMC3584156

[B42] Duncan C. J. Hill A. V. (2011). What is the efficacy of the RTS, S malaria vaccine? *BMJ* 343 d7728. 10.1136/bmj.d7728 PMC3287289 22167776

[B43] Elliott S. R. Kuns R. D. Good M. F. (2005). Heterologous immunity in the absence of variant-specific antibodies after exposure to subpatent infection with blood-stage malaria. *Infect. Immun.* 73 2478–2485 10.1128/IAI.73.4.2478-2485.2005 15784594 PMC1087398

[B44] Epstein J. E. Tewari K. Lyke K. E. Sim B. K. Billingsley P. F. Laurens M. B. (2011). Live attenuated malaria vaccine designed to protect through hepatic CD8(+) T cell immunity. *Science* 334 475–480 10.1126/science.1211548 21903775

[B45] Florens L. Liu X. Wang Y. Yang S. Schwartz O. Peglar M. (2004). Proteomics approach reveals novel proteins on the surface of malaria-infected erythrocytes. *Mol. Biochem. Parasitol.* 135 1–11 10.1016/j.molbiopara.2003.12.007 15287581

[B46] Florens L. Washburn M. P. Raine J. D. Anthony R. M. Grainger M. Haynes J. D. (2002). A proteomic view of the *Plasmodium falciparum* life cycle. *Nature* 419 520–526 10.1038/nature01107 12368866

[B47] Florent I. Charneau S. Grellier P. (2004). *Plasmodium falciparum* genes differentially expressed during merozoite morphogenesis. *Mol. Biochem. Parasitol.* 135 143–148 10.1016/j.molbiopara.2003.12.010 15287595

[B48] Florent I. Porcel B. M. Guillaume E. Da Silva C. Artiguenave F. Marechal E. (2009). A *Plasmodium falciparum* FcB1-schizont-EST collection providing clues to schizont specific gene structure and polymorphism. *BMC Genom.* 10:235 10.1186/1471-2164-10-235 PMC2695484 19454033

[B49] Friedrich T. C. Valentine L. E. Yant L. J. Rakasz E. G. Piaskowski S. M. Furlott J. R. (2007). Subdominant CD8+ T-cell responses are involved in durable control of AIDS virus replication. *J. Virol.* 81 3465–3476 10.1128/JVI.02392-06 17251286 PMC1866056

[B50] Friesen J. Silvie O. Putrianti E. D. Hafalla J. C. Matuschewski K. Borrmann S. (2010). Natural immunization against malaria: causal prophylaxis with antibiotics. *Sci. Transl. Med.* 2 40ra49. 10.1126/scitranslmed.3001058 20630856

[B51] Gardner M. J. Hall N. Fung E. White O. Berriman M. Hyman R. W. (2002). Genome sequence of the human malaria parasite *Plasmodium falciparum*. *Nature* 419 498–511 10.1038/nature01097 12368864 PMC3836256

[B52] Good M. F. Doolan D. L. (2010). Malaria vaccine design: immunological considerations. *Immunity* 33 555–566 10.1016/j.immuni.2010.10.005 21029965

[B53] Good M. F. Kumar S. Miller L. H. (1988). The real difficulties for malaria sporozoite vaccine development: nonresponsiveness and antigenic variation. *Immunol. Today* 9 351–355 10.1016/0167-5699(88)91336-9 3076406

[B54] Good M. F. Reiman J. M. Rodriguez I. B. Ito K. Yanow S. K. El-Deeb I. M. (2013). Cross-species malaria immunity induced by chemically attenuated parasites. *J. Clin. Invest.* 123 3353–3362 10.1172/JCI66634 PMC4011145 23863622

[B55] Gruner A. C. Mauduit M. Tewari R. Romero J. F. Depinay N. Kayibanda M. (2007). Sterile protection against malaria is independent of immune responses to the circumsporozoite protein. *PLoS ONE* 2:e1371 10.1371/journal.pone.0001371 PMC2147056 18159254

[B56] Gunasekera A. M. Patankar S. Schug J. Eisen G. Kissinger J. Roos D. (2004). Widespread distribution of antisense transcripts in the *Plasmodium falciparum* genome. *Mol. Biochem. Parasitol.* 136 35–42 10.1016/j.molbiopara.2004.02.007 15138065

[B57] Gunasekera A. M. Patankar S. Schug J. Eisen G. Wirth D. F. (2003). Drug-induced alterations in gene expression of the asexual blood forms of *Plasmodium falciparum*. *Mol. Microbiol.* 50 1229–1239 10.1046/j.1365-2958.2003.03787.x 14622411

[B58] Gwadz R. W. Cochrane A. H. Nussenzweig V. Nussenzweig R. S. (1979). Preliminary studies on vaccination of rhesus monkeys with irradiated sporozoites of *Plasmodium knowlesi* and characterization of surface antigens of these parasites. *Bull. World Health Organ.* 57(Suppl. 1), 165–173. 120766 PMC2395714

[B59] Hafalla J. C. Bauza K. Friesen J. Gonzalez-Aseguinolaza G. Hill A. V. Matuschewski K. (2013). Identification of targets of CD8(+) T cell responses to malaria liver stages by genome-wide epitope profiling. *PLoS Pathog.* 9:e1003303 10.1371/journal.ppat.1003303 PMC3649980 23675294

[B60] Hall B. F. Fauci A. S. (2009). Malaria control, elimination, and eradication: the role of the evolving biomedical research agenda. *J. Infect. Dis.* 200 1639–1643 10.1086/646611 19877843

[B61] Hall N. Karras M. Raine J. D. Carlton J. M. Kooij T. W. Berriman M. (2005). A comprehensive survey of the *Plasmodium* life cycle by genomic, transcriptomic, and proteomic analyses. *Science* 307 82–86 10.1126/science.1103717 15637271

[B62] Hirunpetcharat C. Good M. F. (1998). Deletion of *Plasmodium berghei*-specific CD4+ T cells adoptively transferred into recipient mice after challenge with homologous parasite. *Proc. Natl. Acad. Sci. U.S.A.* 95 1715–1720 10.1073/pnas.95.4.1715 9465082 PMC19161

[B63] Hoffman S. L. Goh L. M. Luke T. C. Schneider I. Le T. P. Doolan D. L. (2002). Protection of humans against malaria by immunization with radiation-attenuated *Plasmodium falciparum* sporozoites. *J. Infect. Dis.* 185 1155–1164 10.1086/339409 11930326

[B64] Jenner E. (1798). *An Inquiry into Causes and Effects of Variolae Vaccinae, a Disease, Discovered in some of the Western Countries of England, Particularly Gloucestershire, and know by the name of Cow Pox*. London: Printed by Sampson Low.

[B65] Kariu T. Ishino T. Yano K. Chinzei Y. Yuda M. (2006). CelTOS, a novel malarial protein that mediates transmission to mosquito and vertebrate hosts. *Mol. Microbiol.* 59 1369–1379 10.1111/j.1365-2958.2005.05024.x 16468982

[B66] Khan S. M. Franke-Fayard B. Mair G. R. Lasonder E. Janse C. J. Mann M. (2005). Proteome analysis of separated male and female gametocytes reveals novel sex-specific *Plasmodium biology*. *Cell* 121 675–687 10.1016/j.cell.2005.03.027 15935755

[B67] Kirkman L. A. Deitsch K. W. (2012). Antigenic variation and the generation of diversity in malaria parasites. *Curr. Opin. Microbiol.* 15 456–462 10.1016/j.mib.2012.03.003 22503815 PMC3399988

[B68] Kloverpris H. Karlsson I. Bonde J. Thorn M. Vinner L. Pedersen A. E. (2009). Induction of novel CD8+ T-cell responses during chronic untreated HIV-1 infection by immunization with subdominant cytotoxic T-lymphocyte epitopes. *AIDS* 23 1329–1340 10.1097/QAD.0b013e32832d9b00 19528789

[B69] Klysik J. (2001). Concept of immunomics: a new frontier in the battle for gene function? *Acta Biotheor.* 49 191–202 10.1023/A:1011901410166 11558896

[B70] Kumar K. A. Sano G. Boscardin S. Nussenzweig R. S. Nussenzweig M. C. Zavala F. (2006). The circumsporozoite protein is an immunodominant protective antigen in irradiated sporozoites. *Nature* 444 937–940 10.1038/nature05361 17151604

[B71] Labaied M. Harupa A. Dumpit R. F. Coppens I. Mikolajczak S. A. Kappe S. H. (2007). *Plasmodium yoelii* sporozoites with simultaneous deletion of P52 and P36 are completely attenuated and confer sterile immunity against infection. *Infect. Immun.* 75 3758–3768 10.1128/IAI.00225-07 17517871 PMC1951999

[B72] Langhorne J. Ndungu F. M. Sponaas A. M. Marsh K. (2008). Immunity to malaria: more questions than answers. *Nat. Immunol.* 9 725–732 10.1038/ni.f.205 18563083

[B73] Lasonder E. Ishihama Y. Andersen J. S. Vermunt A. M. Pain A. Sauerwein R. W. (2002). Analysis of the *Plasmodium falciparum* proteome by high-accuracy mass spectrometry. *Nature* 419 537–542 10.1038/nature01111 12368870

[B74] Lasonder E. Janse C. J. van Gemert G. J. Mair G. R. Vermunt A. M. Douradinha B. G. (2008). Proteomic profiling of *Plasmodium sporozoite* maturation identifies new proteins essential for parasite development and infectivity. *PLoS Pathog.* 4:e1000195 10.1371/journal.ppat.1000195 PMC2570797 18974882

[B75] Le Roch K. G. Johnson J. R. Florens L. Zhou Y. Santrosyan A. Grainger M. (2004). Global analysis of transcript and protein levels across the *Plasmodium falciparum* life cycle. *Genome Res.* 14 2308–2318 10.1101/gr.2523904 15520293 PMC525690

[B76] Le Roch K. G. Zhou Y. Blair P. L. Grainger M. Moch J. K. Haynes J. D. (2003). Discovery of gene function by expression profiling of the malaria parasite life cycle. *Science* 301 1503–1508 10.1126/science.1087025 12893887

[B77] Limbach K. Aguiar J. Gowda K. Patterson N. Abot E. Sedegah M. (2011). Identification of two new protective pre-erythrocytic malaria vaccine antigen candidates. *Malar. J.* 10 65 10.1186/1475-2875-10-65 PMC3073953 21410955

[B78] Lindner S. E. Swearingen K. E. Harupa A. Vaughan A. M. Sinnis P. Moritz R. L. (2013). Total and putative surface proteomics of malaria parasite salivary gland sporozoites. *Mol. Cell. Proteom.* 12 1127–1143 10.1074/mcp.M112.024505 PMC3650326 23325771

[B79] Liu W. Li Y. Learn G. H. Rudicell R. S. Robertson J. D. Keele B. F. (2010). Origin of the human malaria parasite *Plasmodium falciparum* in gorillas. *Nature* 467 420–425 10.1038/nature09442 20864995 PMC2997044

[B80] Llinas M. Bozdech Z. Wong E. D. Adai A. T. DeRisi J. L. (2006). Comparative whole genome transcriptome analysis of three *Plasmodium falciparum* strains. *Nucleic Acids Res.* 34 1166–1173 10.1093/nar/gkj517 16493140 PMC1380255

[B81] Lopez-Barragan M. J. Lemieux J. Quinones M. Williamson K. C. Molina-Cruz A. Cui K. (2011). Directional gene expression and antisense transcripts in sexual and asexual stages of *Plasmodium falciparum*. *BMC Genom.* 12:587 10.1186/1471-2164-12-587 PMC3266614 22129310

[B82] Mackinnon M. J. Marsh K. (2010). The selection landscape of malaria parasites. *Science* 328 866–871 10.1126/science.1185410 20466925

[B83] Matuschewski K. Hafalla J. C. Borrmann S. Friesen J. (2011). Arrested *Plasmodium* liver stages as experimental anti-malaria vaccines. *Hum. Vaccin.* 7(Suppl.), 16–21 10.4161/hv.7.0.14557 21266857

[B84] Mauduit M. Tewari R. Depinay N. Kayibanda M. Lallemand E. Chavatte J. M. (2010). Minimal role for the circumsporozoite protein in the induction of sterile immunity by vaccination with live rodent malaria sporozoites. *Infect. Immun.* 78 2182–2188 10.1128/IAI.01415-09 20194600 PMC2863544

[B85] Menard R. (2005). Medicine: knockout malaria vaccine? *Nature* 433 113–114 10.1038/433113a 15650722

[B86] Mikolajczak S. A. Lakshmanan V. Fishbaugher M. Camargo N. Harupa A. Kaushansky A. (2014). A next-generation genetically attenuated *Plasmodium falciparum* parasite created by triple gene deletion. *Mol. Ther.* 22 1707–1715 10.1038/mt.2014.85 24827907 PMC4435496

[B87] Mikolajczak S. A. Silva-Rivera H. Peng X. Tarun A. S. Camargo N. Jacobs-Lorena V. (2008). Distinct malaria parasite sporozoites reveal transcriptional changes that cause differential tissue infection competence in the mosquito vector and mammalian host. *Mol. Cell. Biol.* 28 6196–6207 10.1128/MCB.00553-08 18710954 PMC2577418

[B88] Mishra S. Rai U. Shiratsuchi T. Li X. Vanloubbeeck Y. Cohen J. (2011). Identification of non-CSP antigens bearing CD8 epitopes in mice immunized with irradiated sporozoites. *Vaccine* 29 7335–7342 10.1016/j.vaccine.2011.07.081 21807053 PMC3603353

[B89] Moorthy V. S. Kieny M. P. (2010). Reducing empiricism in malaria vaccine design. *Lancet Infect. Dis.* 10 204–211 10.1016/S1473-3099(09)70329-9 20185099

[B90] Mueller A. K. Labaied M. Kappe S. H. Matuschewski K. (2005). Genetically modified *Plasmodium parasites* as a protective experimental malaria vaccine. *Nature* 433 164–167 10.1038/nature03188 15580261

[B91] Nahrendorf W. Scholzen A. Bijker E. M. Teirlinck A. C. Bastiaens G. J. Schats R. (2014). Memory B-cell and antibody responses induced by *Plasmodium falciparum* sporozoite immunization. *J. Infect. Dis.* 10.1093/infdis/jiu354 [Epub ahead of print]. PMC4241945 24970846

[B92] Nganou-Makamdop K. Sauerwein R. W. (2013). Liver or blood-stage arrest during malaria sporozoite immunization: the later the better? *Trends Parasitol.* 29 304–310 10.1016/j.pt.2013.03.008 23608185

[B93] Nunes J. K. Cardenas V. Loucq C. Maire N. Smith T. Shaffer C. (2013). Modeling the public health impact of malaria vaccines for developers and policymakers. *BMC Infect. Dis.* 13:295 10.1186/1471-2334-13-295 PMC3711926 23815273

[B94] Nussenzweig R. S. Vanderberg J. Most H. Orton C. (1967). Protective immunity produced by the injection of X-irradiated sporozoites of *Plasmodium berghei*. *Nature* 216 160–162 10.1038/216160a0 6057225

[B95] Oehring S. C. Woodcroft B. J. Moes S. Wetzel J. Dietz O. Pulfer A. (2012). Organellar proteomics reveals hundreds of novel nuclear proteins in the malaria parasite *Plasmodium falciparum*. *Genome Biol.* 13 R108. 10.1186/gb-2012-13-11-r108 PMC4053738 23181666

[B96] Okell L. C. Ghani A. C. Lyons E. Drakeley C. J. (2009). Submicroscopic infection in *Plasmodium falciparum*-endemic populations: a systematic review and meta-analysis. *J. Infect. Dis.* 200 1509–1517 10.1086/644781 19848588

[B97] Olotu A. Fegan G. Wambua J. Nyangweso G. Awuondo K. O. Leach A. (2013). Four-year efficacy of RTS,S/AS01E and its interaction with malaria exposure. *N. Engl. J. Med.* 368 1111–1120 10.1056/NEJMoa1207564 23514288 PMC5156295

[B98] Orjih A. U. Cochrane A. H. Nussenzweig R. S. (1982). Comparative studies on the immunogenicity of infective and attenuated sporozoites of *Plasmodium berghei*. *Trans. R. Soc. Trop. Med. Hyg.* 76 57–61 10.1016/0035-9203(82)90019-0 7043807

[B99] Osier F. H. Fegan G. Polley S. D. Murungi L. Verra F. Tetteh K. K. (2008). Breadth and magnitude of antibody responses to multiple *Plasmodium falciparum* merozoite antigens are associated with protection from clinical malaria. *Infect. Immun.* 76 2240–2248 10.1128/IAI.01585-07 18316390 PMC2346713

[B100] Otto T. D. Wilinski D. Assefa S. Keane T. M. Sarry L. R. Bohme U. (2010). New insights into the blood-stage transcriptome of *Plasmodium falciparum* using RNA-Seq. *Mol. Microbiol.* 76 12–24 10.1111/j.1365-2958.2009.07026.x 20141604 PMC2859250

[B101] Owusu-Agyei S. Koram K. A. Baird J. K. Utz G. C. Binka F. N. Nkrumah F. K. (2001). Incidence of symptomatic and asymptomatic *Plasmodium falciparum* infection following curative therapy in adult residents of northern Ghana. *Am. J. Trop. Med. Hyg.* 65 197–203. 11561704 10.4269/ajtmh.2001.65.197

[B102] Pain A. Bohme U. Berry A. E. Mungall K. Finn R. D. Jackson A. P. (2008). The genome of the simian and human malaria parasite *Plasmodium knowlesi*. *Nature* 455 799–803 10.1038/nature07306 18843368 PMC2656934

[B103] Patankar S. Munasinghe A. Shoaibi A. Cummings L. M. Wirth D. F. (2001). Serial analysis of gene expression in *Plasmodium falciparum* reveals the global expression profile of erythrocytic stages and the presence of anti-sense transcripts in the malarial parasite. *Mol. Biol. Cell* 12 3114–3125 10.1091/mbc.12.10.3114 11598196 PMC60160

[B104] Pierce S. K. Miller L. H. (2009). World Malaria Day 2009: what malaria knows about the immune system that immunologists still do not. *J. Immunol.* 182 5171–5177 10.4049/jimmunol.0804153 19380759 PMC2779769

[B105] Plowe C. V. Alonso P. Hoffman S. L. (2009). The potential role of vaccines in the elimination of falciparum malaria and the eventual eradication of malaria. *J. Infect. Dis.* 200 1646–1649 10.1086/646613 19877844

[B106] Pombo D. J. Lawrence G. Hirunpetcharat C. Rzepczyk C. Bryden M. Cloonan N. (2002). Immunity to malaria after administration of ultra-low doses of red cells infected with *Plasmodium falciparum*. *Lancet* 360 610–617 10.1016/S0140-6736(02)09784-2 12241933

[B107] Purcell L. A. Wong K. A. Yanow S. K. Lee M. Spithill T. W. Rodriguez A. (2008a). Chemically attenuated *Plasmodium sporozoites* induce specific immune responses, sterile immunity and cross-protection against heterologous challenge. *Vaccine* 26 4880–4884 10.1016/j.vaccine.2008.07.017 18672017 PMC2560175

[B108] Purcell L. A. Yanow S. K. Lee M. Spithill T. W. Rodriguez A. (2008b). Chemical attenuation of *Plasmodium berghei* sporozoites induces sterile immunity in mice. *Infect. Immun.* 76 1193–1199 10.1128/IAI.01399-07 18174336 PMC2258828

[B109] Putrianti E. D. Silvie O. Kordes M. Borrmann S. Matuschewski K. (2009). Vaccine-like immunity against malaria by repeated causal-prophylactic treatment of liver-stage *Plasmodium parasites*. *J. Infect. Dis.* 199 899–903 10.1086/597121 19434915

[B110] Roberts L. Enserink M. (2007). Malaria. Did they really say... eradication? *Science* 318 1544–1545 10.1126/science.318.5856.1544 18063766

[B111] Roestenberg M. McCall M. Hopman J. Wiersma J. Luty A. J. van Gemert G. J. (2009). Protection against a malaria challenge by sporozoite inoculation. *N. Engl. J. Med.* 361 468–477 10.1056/NEJMoa0805832 19641203

[B112] Roestenberg M. Teirlinck A. C. McCall M. B. Teelen K. Makamdop K. N. Wiersma J. (2011). Long-term protection against malaria after experimental sporozoite inoculation: an open-label follow-up study. *Lancet* 377 1770–1776 10.1016/S0140-6736(11)60360-7 21514658

[B113] Roobsoong W. Roytrakul S. Sattabongkot J. Li J. Udomsangpetch R. Cui L. (2011). Determination of the *Plasmodium vivax* schizont stage proteome. *J. Proteom.* 74 1701–1710 10.1016/j.jprot.2011.03.035 PMC3156846 21515433

[B114] Rovira-Graells N. Gupta A. P. Planet E. Crowley V. M. Mok S. Ribas de Pouplana L. (2012). Transcriptional variation in the malaria parasite *Plasmodium falciparum*. *Genome Res.* 22 925–938 10.1101/gr.129692.111 22415456 PMC3337437

[B115] Ruckwardt T. J. Luongo C. Malloy A. M. Liu J. Chen M. Collins P. L. (2010). Responses against a subdominant CD8+ T cell epitope protect against immunopathology caused by a dominant epitope. *J. Immunol.* 185 4673–4680 10.4049/jimmunol.1001606 20833834 PMC4144756

[B116] Rueckert C. Guzman C. A. (2012). Vaccines: from empirical development to rational design. *PLoS Pathog.* 8:e1003001 10.1371/journal.ppat.1003001 PMC3493475 23144616

[B117] Sabchareon A. Burnouf T. Ouattara D. Attanath P. Bouharoun-Tayoun H. Chantavanich P. (1991). Parasitologic and clinical human response to immunoglobulin administration in *Falciparum malaria*. *Am. J. Trop. Med. Hyg.* 45 297–308. 1928564 10.4269/ajtmh.1991.45.297

[B118] Sacci J. B. Jr. Ribeiro J. M. Huang F. Alam U. Russell J. A. Blair P. L. (2005). Transcriptional analysis of in vivo *Plasmodium yoelii* liver stage gene expression. *Mol. Biochem. Parasitol.* 142 177–183 10.1016/j.molbiopara.2005.03.018 15876462

[B119] Sam-Yellowe T. Y. Florens L. Wang T. Raine J. D. Carucci D. J. Sinden R. (2004). Proteome analysis of rhoptry-enriched fractions isolated from *Plasmodium merozoites*. *J. Proteome Res.* 3 995–1001 10.1021/pr049926m 15473688

[B120] Schmidt N. W. Butler N. S. Badovinac V. P. Harty J. T. (2010). Extreme CD8 T cell requirements for anti-malarial liver-stage immunity following immunization with radiation attenuated sporozoites. *PLoS Pathog.* 6:e1000998 10.1371/journal.ppat.1000998 PMC2904779 20657824

[B121] Schmidt N. W. Podyminogin R. L. Butler N. S. Badovinac V. P. Tucker B. J. Bahjat K. S. (2008). Memory CD8 T cell responses exceeding a large but definable threshold provide long-term immunity to malaria. *Proc. Natl. Acad. Sci. U.S.A.* 105 14017–14022 10.1073/pnas.0805452105 18780790 PMC2544571

[B122] Schofield L. Villaquiran J. Ferreira A. Schellekens H. Nussenzweig R. Nussenzweig V. (1987). Gamma interferon, CD8+ T cells and antibodies required for immunity to malaria sporozoites. *Nature* 330 664–666 10.1038/330664a0 3120015

[B123] Schussek S. Trieu A. Doolan D. L. (2014). Genome- and proteome-wide screening strategies for antigen discovery and immunogen design. *Biotechnol. Adv.* 32 403–414 10.1016/j.biotechadv.2013.12.006 24389084

[B124] Schwartz L. Brown G. V. Genton B. Moorthy V. S. (2012). A review of malaria vaccine clinical projects based on the WHO rainbow table. *Malar. J.* 11 11 10.1186/1475-2875-11-11 PMC3286401 22230255

[B125] Seder R. A. Chang L. J. Enama M. E. Zephir K. L. Sarwar U. N. Gordon I. J. (2013). Protection against malaria by intravenous immunization with a nonreplicating sporozoite vaccine. *Science* 341 1359–1365 10.1126/science.1241800 23929949

[B126] Sercarz E. E. Lehmann P. V. Ametani A. Benichou G. Miller A. Moudgil K. (1993). Dominance and crypticity of T cell antigenic determinants. *Annu. Rev. Immunol.* 11 729–766 10.1146/annurev.iy.11.040193.003501 7682817

[B127] Sherman I. W. (2009). *The Elusive Malaria Vaccine: Miracle or Mirage?* Washington, DC: American Society for Microbiology.

[B128] Siau A. Silvie O. Franetich J. F. Yalaoui S. Marinach C. Hannoun L. (2008). Temperature shift and host cell contact up-regulate sporozoite expression of *Plasmodium falciparum* genes involved in hepatocyte infection. *PLoS Pathog.* 4:e1000121 10.1371/journal.ppat.1000121 PMC2488394 18688281

[B129] Silvestrini F. Lasonder E. Olivieri A. Camarda G. van Schaijk B. Sanchez M. (2010). Protein export marks the early phase of gametocytogenesis of the human malaria parasite *Plasmodium falciparum*. *Mol. Cell. Proteom.* 9 1437–1448 10.1074/mcp.M900479-MCP200 PMC2938084 20332084

[B130] Spring M. Murphy J. Nielsen R. Dowler M. Bennett J. W. Zarling S. (2013). First-in-human evaluation of genetically attenuated *Plasmodium falciparum* sporozoites administered by bite of *Anopheles mosquitoes* to adult volunteers. *Vaccine* 31 4975–4983 10.1016/j.vaccine.2013.08.007 24029408

[B131] Stephens R. Albano F. R. Quin S. Pascal B. J. Harrison V. Stockinger B. (2005). Malaria-specific transgenic CD4+ T cells protect immunodeficient mice from lethal infection and demonstrate requirement for a protective threshold of antibody production for parasite clearance. *Blood* 106 1676–1684 10.1182/blood-2004-10-4047 15890689

[B132] Tachibana S. Sullivan S. A. Kawai S. Nakamura S. Kim H. R. Goto N. (2012). *Plasmodium cynomolgi* genome sequences provide insight into *Plasmodium vivax* and the monkey malaria clade. *Nat. Genet.* 44 1051–1055 10.1038/ng.2375 22863735 PMC3759362

[B133] Takala S. L. Plowe C. V. (2009). Genetic diversity and malaria vaccine design, testing and efficacy: preventing and overcoming ‘vaccine resistant malaria.’ *Parasite Immunol.* 31 560–573 10.1111/j.1365-3024.2009.01138.x 19691559 PMC2730200

[B134] Tarun A. S. Peng X. Dumpit R. F. Ogata Y. Silva-Rivera H. Camargo N. (2008). A combined transcriptome and proteome survey of malaria parasite liver stages. *Proc. Natl. Acad. Sci. U.S.A.* 105 305–310 10.1073/pnas.0710780104 18172196 PMC2224207

[B135] Taylor S. M. Cerami C. Fairhurst R. M. (2013). Hemoglobinopathies: slicing the Gordian knot of *Plasmodium falciparum* malaria pathogenesis. *PLoS Pathog.* 9:e1003327 10.1371/journal.ppat.1003327 PMC3656091 23696730

[B136] Tran T. M. Li S. Doumbo S. Doumtabe D. Huang C. Y. Dia S. (2013). An intensive longitudinal cohort study of Malian children and adults reveals no evidence of acquired immunity to *Plasmodium falciparum* infection. *Clin. Infect. Dis.* 57 40–47 10.1093/cid/cit174 23487390 PMC3669526

[B137] Trieu A. Kayala M. A. Burk C. Molina D. M. Freilich D. A. Richie T. L. (2011). Sterile protective immunity to malaria is associated with a panel of novel P. falciparum antigens. *Mol. Cell. Proteomics* 10 M111 007948 10.1074/mcp.M111.007948 PMC3186199 21628511

[B138] Tsuji M. (2010). A retrospective evaluation of the role of T cells in the development of malaria vaccine. *Exp. Parasitol.* 126 421–425 10.1016/j.exppara.2009.11.009 19944099 PMC3603350

[B139] Tuells J. (2012). Vaccinology: the name, the concept, the adjectives. *Vaccine* 30 5491–5495 10.1016/j.vaccine.2012.06.059 22766245

[B140] Vignali M. Armour C. D. Chen J. Morrison R. Castle J. C. Biery M. C. (2011). NSR-seq transcriptional profiling enables identification of a gene signature of *Plasmodium falciparum* parasites infecting children. *J. Clin. Invest.* 121 1119–1129 10.1172/JCI43457 21317536 PMC3046638

[B141] Wang C. W. Mwakalinga S. B. Sutherland C. J. Schwank S. Sharp S. Hermsen C. C. (2010). Identification of a major rif transcript common to gametocytes and sporozoites of *Plasmodium falciparum*. *Malar. J.* 9 147 10.1186/1475-2875-9-147 PMC2890677 20509952

[B142] Watanabe J. Wakaguri H. Sasaki M. Suzuki Y. Sugano S. (2007). Comparasite: a database for comparative study of transcriptomes of parasites defined by full-length cDNAs. *Nucleic Acids Res.* 35 D431–D438 10.1093/nar/gkl1039 17151081 PMC1781114

[B143] Weiss W. R. Jiang C. G. (2012). Protective CD8+ T lymphocytes in primates immunized with malaria sporozoites. *PLoS ONE* 7:e31247 10.1371/journal.pone.0031247 PMC3280278 22355349

[B144] Westenberger S. J. McClean C. M. Chattopadhyay R. Dharia N. V. Carlton J. M. Barnwell J. W. (2010). A systems-based analysis of *Plasmodium vivax* lifecycle transcription from human to mosquito. *PLoS Negl. Trop. Dis.* 4:e653 10.1371/journal.pntd.0000653 PMC2850316 20386602

[B145] Winzeler E. A. (2006). Applied systems biology and malaria. *Nat. Rev. Microbiol.* 4 145–151 10.1038/nrmicro1327 16362033

[B146] World Health Organization [WHO]. (2006). *State of the Art of New Vaccine Research and Development.* Geneva: World Health Organization.

[B147] World Health Organization [WHO]. (2012). *Global Vaccine Action Plan 2011 –2020.* Geneva: WHO Press.

[B148] World Health Organization [WHO]. (2014a). *The 10 Leading Causes of Death in the World, 2000 and 2012.* Geneva: World Health Organization.

[B149] World Health Organization [WHO]. (2014b). *Malaria Vaccine Rainbow Tables.* Geneva: World Health Organization.

[B150] Xu H. Wipasa J. Yan H. Zeng M. Makobongo M. O. Finkelman F. D. (2002). The mechanism and significance of deletion of parasite-specific CD4(+) T cells in malaria infection. *J. Exp. Med.* 195 881–892 10.1084/jem.20011174 11927632 PMC2193727

[B151] Young J. A. Fivelman Q. L. Blair P. L. de la Vega P. Le Roch K. G. Zhou Y. (2005). The *Plasmodium falciparum* sexual development transcriptome: a microarray analysis using ontology-based pattern identification. *Mol. Biochem. Parasitol.* 143 67–79 10.1016/j.molbiopara.2005.05.007 16005087

[B152] Zepp F. (2010). Principles of vaccine design - lessons from nature. *Vaccine* 28(Suppl. 3), C14–C24 10.1016/j.vaccine.2010.07.020 20713252

[B153] Zhang X. Tolzmann C. A. Melcher M. Haas B. J. Gardner M. J. Smith J. D. (2011). Branch point identification and sequence requirements for intron splicing in *Plasmodium falciparum*. *Eukaryot. Cell* 10 1422–1428 10.1128/EC.05193-11 21926333 PMC3209046

[B154] Zhang Y. Qi Y. Li J. Liu S. Hong L. Lin T. (2012). A new malaria antigen produces partial protection against *Plasmodium yoelii* challenge. *Parasitol. Res.* 110 1337–1345 10.1007/s00436-011-2630-y 21915626 PMC3437252

